# A comprehensive assessment of photosynthetic acclimation to shade in C4 grass (*Cynodon dactylon* (L.) Pers.)

**DOI:** 10.1186/s12870-024-05242-x

**Published:** 2024-06-21

**Authors:** Guangyang Wang, Jinyan Mao, Mingxia Ji, Wei Wang, Jinmin Fu

**Affiliations:** 1 Coastal Salinity Tolerant Grass Engineering and Technology Research Center, Ludong University, Yantai, 264025 Shandong China; 2 College of Agriculture, Ludong University, Yantai, 264025 Shandong China

**Keywords:** Bermudagrass, Shade tolerance, Photosynthetic acclimation, Chlorophyll fluorescence, Carbon and nitrogen metabolism, Reactive oxygen

## Abstract

**Background:**

Light deficit in shaded environment critically impacts the growth and development of turf plants. Despite this fact, past research has predominantly concentrated on shade avoidance rather than shade tolerance. To address this, our study examined the photosynthetic adjustments of Bermudagrass when exposed to varying intensities of shade to gain an integrative understanding of the shade response of C4 turfgrass.

**Results:**

We observed alterations in photosynthetic pigment-proteins, electron transport and its associated carbon and nitrogen assimilation, along with ROS-scavenging enzyme activity in shaded conditions. Mild shade enriched Chl b and LHC transcripts, while severe shade promoted Chl a, carotenoids and photosynthetic electron transfer beyond Q_A_^−^ (ET_0_/RC, φE_0_, Ψ_0_). The study also highlighted differential effects of shade on leaf and root components. For example, Soluble sugar content varied between leaves and roots as shade diminished *SPS*, *SUT1* but upregulated *BAM*. Furthermore, we observed that shading decreased the transcriptional level of genes involving in nitrogen assimilation (e.g. *NR*) and SOD, POD, CAT enzyme activities in leaves, even though it increased in roots.

**Conclusions:**

As shade intensity increased, considerable changes were noted in light energy conversion and photosynthetic metabolism processes along the electron transport chain axis. Our study thus provides valuable theoretical groundwork for understanding how C4 grass acclimates to shade tolerance.

**Supplementary Information:**

The online version contains supplementary material available at 10.1186/s12870-024-05242-x.

## Background

Light plays a pivotal role in plant growth and development. It provides solar energy for photosynthesis, and doubles as an environmental signal, orchestrating morphological and physiological trade-offs throughout the plants’ cycle [1]. However, shade conditions pose a significant barrier in agricultural production [2] and urban greening [3]. Lower vegetation in the intercropping or vertical planting system particularly receives limited light [4, 5], especially ground covers and lawns [6–8].

Filtered by the upper plants, the light intensity at the bottom is reduced. Red (R, λ = 600–700 nm) and blue (B, λ = 400 ~ 500 nm) light are primarily captured by chloroplasts, while far red light (FR, λ = 700 ~ 800 nm) is partially preserved due to reflection of surroundings [8, 9]. This disproportionate reduction results in a depressed R: FR ratio and low B, which photoreceptors perceive as shade signals, leading to two contending strategies: shade avoiding or shade tolerant [10]. The shade-avoidance syndrome (SAS), characterized by rapid elongation of stems and petioles and accelerated flowering, has been extensively studied [11–14]. However, this strategy is limited to plants of similar stature situated in open habitats. For understory plants, the height disparity renders elongation ineffective in escaping shade [15]. Instead, these plants have evolved shade-tolerant strategies, suppressing SAS and reallocating resources towards optimizing photosynthesis and bolstering physical defenses [16–18]. Despite this, our understanding of shade tolerance remains fragmented [15, 19], particularly concerning understory herbs [20, 21], a topic which has so far received limited attention.

Given the partial overlapped of shading perception and response in both shade and non-shade plants [22, 23], it was considered that molecular regulatory components shared between shade tolerance and shade avoidance. The divergence in strategy, however, arises from different signal transduction pathways. Within molecular cascades of shade response, Phytochrome-Interacting Factors (PIF4, PIF5, PIF7) act as core signaling hubs coordinating the bulk of the downstream events [2, 13]. At low R: FR ratio, PIF degradation slows on account of impaired phosphorylation via the inactivation of phyB [24]. Resultantly, an accumulation of PIFs, particularly PIF7 [25, 26], triggers the auxin network, thereby prompting elongation [27]. Furthermore, cryptochromes (cry1 and cry2) perceive reduced blue light, and cry inactivation enhances PIF abundance (PIF4, PIF5) [28, 29]. Notably, PIF activity is moderated by inhibitors in a feedback-regulated method, including HFR1, PAR1, PAR2 [30], and DELLA protein [31]. Antagonistic factors that obstruct the regulatory pathway of the Shade Avoidance Syndrome (SAS) may hint at shade tolerance mechanisms [9].

In response to low light, acclimations also consist of modifications in leaf anatomical structure and chloroplast ultrastructure at cellular and organismal levels. Shade leaves display a higher specific leaf area [32], a larger proportion of spongy tissue [33], and a greater level of grana thylakoid stacking [34] when compared to sun leaves. Additionally, photosynthesis is notably modified under low light [35, 36], with enzyme concentrations in the Calvin-Benson cycle varying with light intensity [37]. The PSII/PSI ratio and LHCII heighten under low light exposure [38]. To acclimate to such low-irradiance environment, post-translational modifications, which mainly occur in the activity of metabolic enzymes, are typically swift and immediate. Examples of these modifications include protein phosphorylation (e.g., Nitrate reductase, EC:1.7.1.1) [39] and sulfhydryl reduction (e.g., Rubisco EC:4.1.1.39, fructose-1,6- bisphosphatase EC:3.1.3.11, sedoheptulose-1,7-bisphosphatase EC:3.1.3.37) [40]. Following environmental perturbation, new equilibrium is established between the photosynthetic electron transport [41], its correlated Calvin-Benson cycle [42], nitrogen assimilation [43], and reactive oxygen species metabolism [44, 45]. This primarily results from the fact that ATP and reducing power needed for these intertwined biochemical processes is drawn from absorbed light energy. A decrease in light brings about more intense competition among these processes.

Bermudagrass (*Cynodon dactylon* (L.) Pers.) is a perennial warm-season (C4) grass of the NAD-ME biochemical subtype [46, 47]. Its extensive usage as turfgrass or forage stem from exceptional resistance to abiotic stresses, yet it proves sensitive to shade [48]. C4 plants, characterized by the CO_2_-concentrating mechanism, demonstrate ecological dominance in warm, high-light environments [49]. However, limited survival of C4 plants in shaded conditions can be attributed to their relatively restrained plasticity and high energy consumption [50, 51]. NAD-ME type, in particular, is the most susceptible to low light among C4 biochemical isoforms [51]. Shading acclimations in C3 photosynthesis have been a focus of extensive studies over the past several decades, whereas comparable research concerning C4 is nearly nonexistent.

In order to investigate long-term photosynthetic acclimation to shade for C4 grass, bermudagrass was subjected to shade via a shading net for a duration of one week. By placing the photosynthetic electron transport at the center of our focus, we linked ROS metabolism with C/N assimilation. This method enabled the tracking of the energy absorption, transport, and utilization process on the thylakoid membrane. Such approach allowed for a comprehensive analysis of the shade-tolerant adaptability of bermudagrass from a holistic perspective.

## Results

### Alterations of photosynthetic pigments under shading conditions

**Fig. 1:**
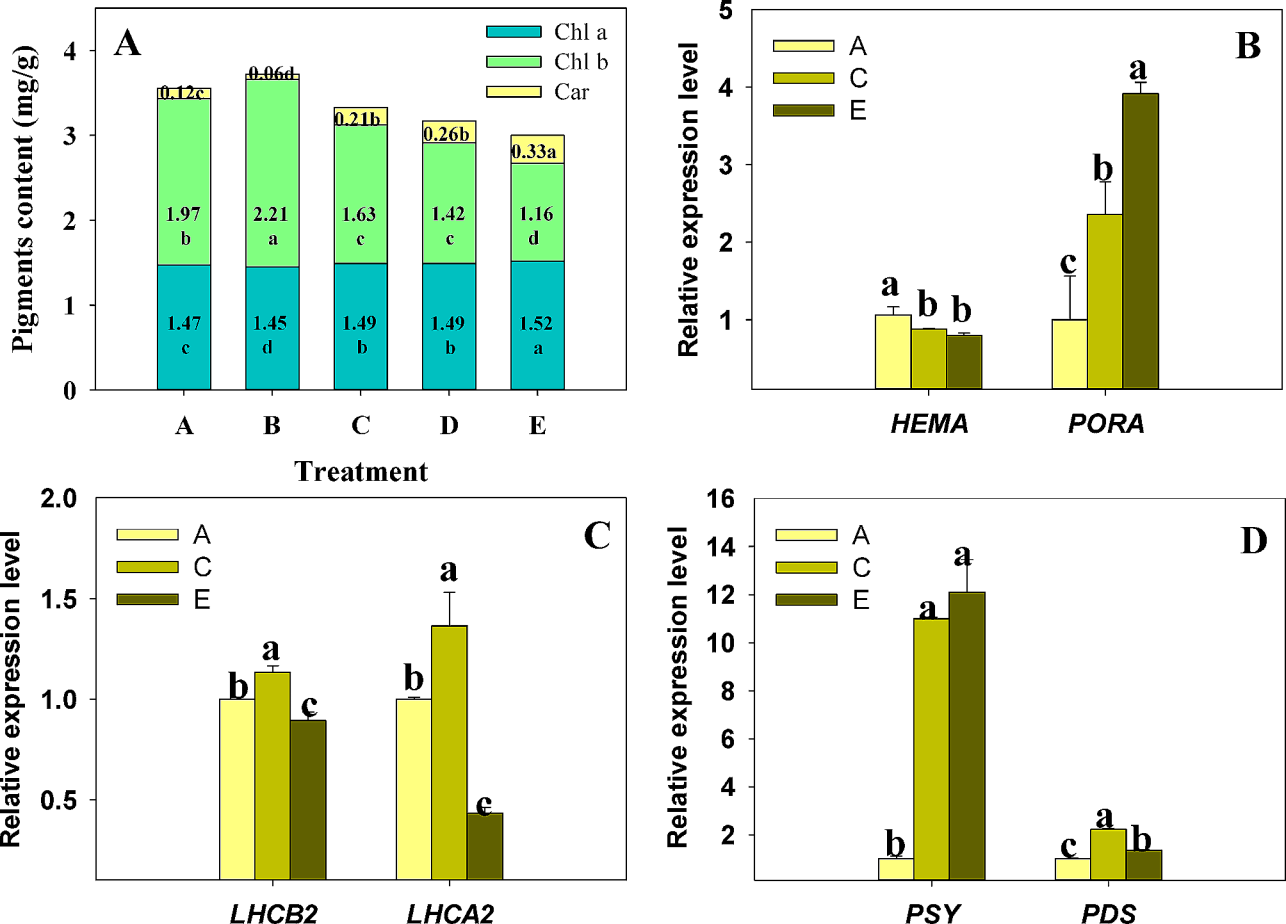
The adjustment of light harvesting capacity of Bermudagrass under shade. (**A**) Photosynthetic pigment content (per gram of fresh weight). The numbers and letters enclosed in boxes refer to the mean values and statistical differences, respectively. The capital letters along the X axis categorize the increasing severity of the shade, ranging from Group A to Group E, indicating the control (100%, 500 µmol m^− 2^ s^− 1^) and subsequent shading treatments of 50% (250 µmol m^− 2^ s^− 1^), 25% (125 µmol m^− 2^ s^− 1^), 12.5% (62.5 µmol m^− 2^ s^− 1^), and 6.25% (31.25 µmol m^− 2^ s^− 1^), respectively. This study examined the transcript levels of key enzymes responsible for chlorophyll (**B**) and carotenoid synthesis (**D**), along with the light-harvesting complex subunits (**C**). The genes encoding these proteins are HEMA (glutamyl-tRNA reductase), PORA (Protochlorophyllide reductase A), PSY (Phytoene synthase), PDS (15-cis-phytoene desaturase), LHCB2 (Photosystem II light harvesting complex gene 2), and LHCA2 (Photosystem I chlorophyll a/b-binding protein 2). The expression levels of these genes were normalized, with the control (Group A) defined as the baseline. The statistical differences were identified using one-way ANOVA and Student-Newman-Keuls multiple range tests, columns marking the letters were used to signify Mean ± SD and statistically significant variation (*P* < 0.05). The study used five biological replicates to ensure accuracy

Gradual shading conditions (Group B to E) led to linear changes in the photosynthetic pigment content within Bermudagrass. Lower light intensity corresponded with decreased total pigment, predominantly due to the significant reduction of chlorophyll b (Fig. 1A). The density of chlorophyll b in group E was almost half that of group B. Notably, chlorophyll b showed an increase in Group B relative to the control (Group A), thereby reducing the ratio of chlorophyll a to chlorophyll b. However, subsequent shade conditions induced an increase in carotenoid content (from 1.63% in group B to 12.27% in group E) and a significant surge in chlorophyll a level. Examination of pigment biosynthesis gene expression levels in selected groups (A, B, and E) provided insights into the observed pigment alterations. Lower light levels induced a down-regulation in the expression of *HEMA*, a gene coding for the enzyme catalyzing the initial step in chlorophyll synthesis (Fig. 1B). This may had led to a decrease in the total amount of chlorophyll. Additionally, it was observed that shading increased the transcript levels of light-harvesting complexes (LHCB2, LHCA2) and enzyme PDS, a crucial player in carotenoid synthesis, under light shade while a decrease was observed under heavy shade (Fig. 1C, D). An interesting observation was a sharp up-regulation of PSY under shading (Fig. 1D). Similarly, PORA’s transcription level saw a 3.9-fold increase in Group E relative to the control (Fig. 1B). All these changes suggest that the alteration in photosynthetic pigment synthesis and pigment-binding protein levels play a significant role in modulating the composition of photosynthetic pigments under different shading conditions.

### Enhanced electron transfer beyond QA- was observed under shaded conditions

**Fig. 2:**
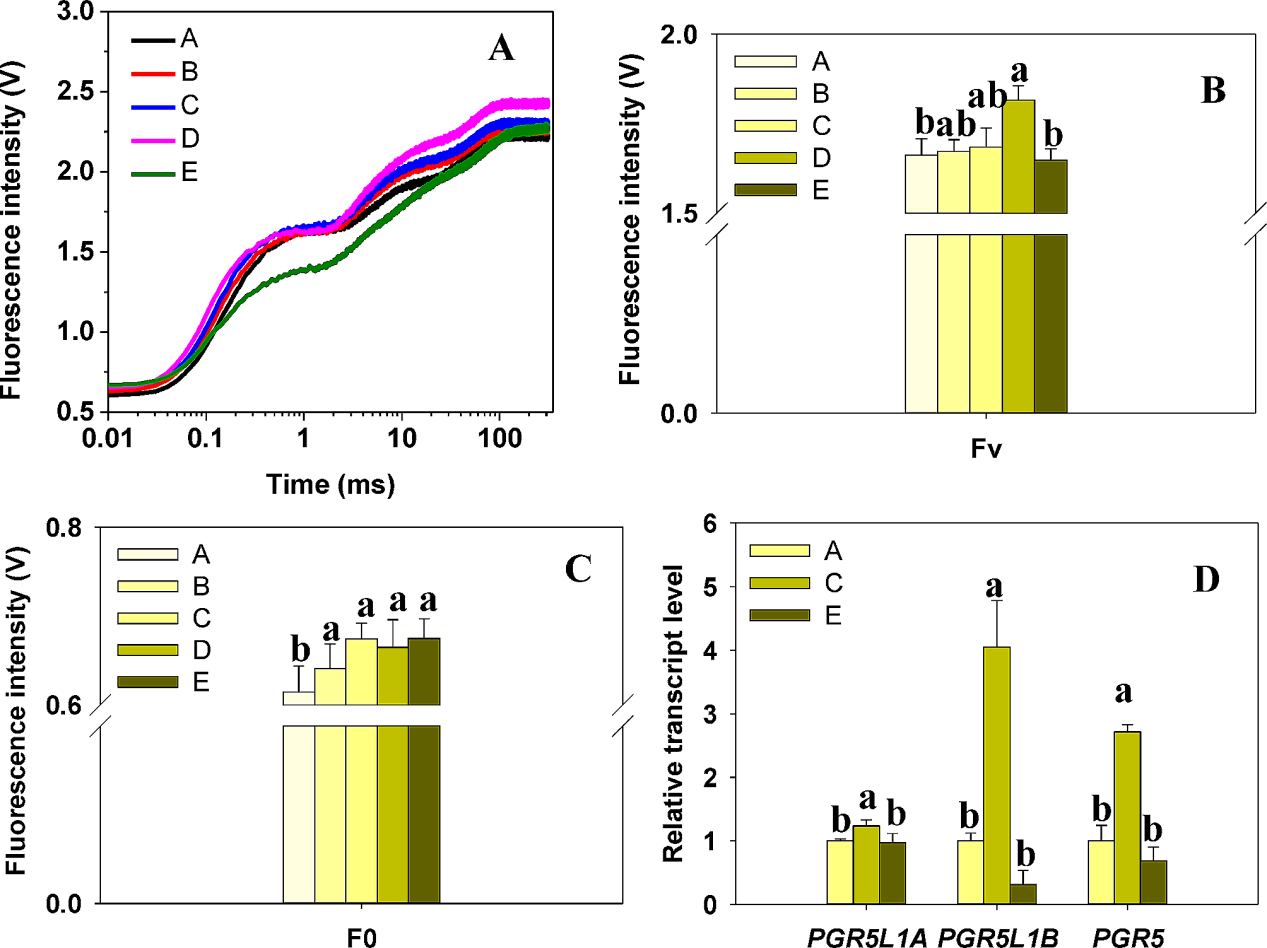
Illustration of shading effects on fast chlorophyll fluorescence transients and cyclic electron transport in Bermudagrass. (**A**) Polyphasic rise of chlorophyll fluorescence under varying shading conditions; (**B, C**) The apex variable fluorescence and initial fluorescence of PSII; (**D**) Transcription levels of *PGR5L1A*, *PGR5L1B*, and *PGR5*, which encode PGR5-like protein 1 A, PGR5-like protein 1B, and Protein PROTON GRADIENT REGULATION 5 respectively. Here, A group is defined as 1. We utilized an ANOVA with Student-Newman-Keuls (*P* < 0.05) in our study. Columns marking the letters were used to signify Mean ± SD and statistically significant variation (*P* < 0.05). Five biological replicates were considered

**Fig. 3:**
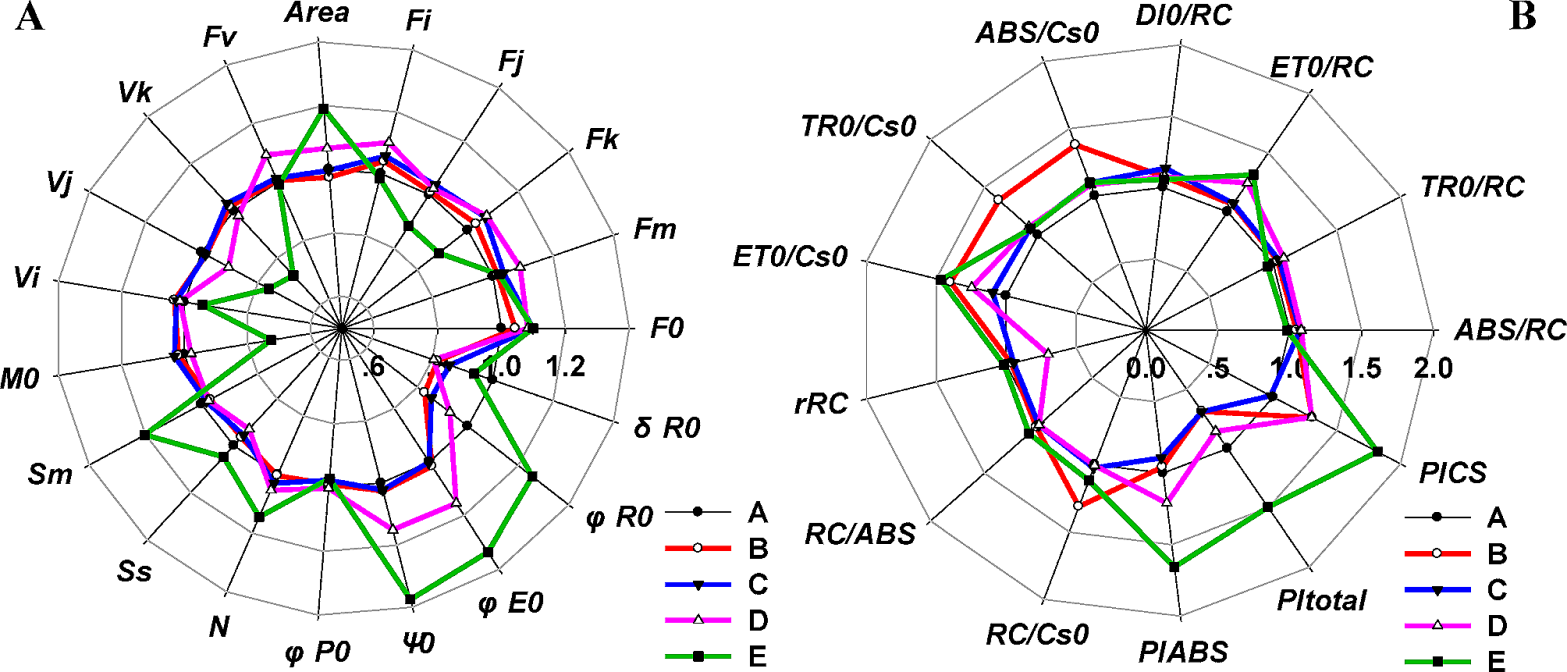
Radar plots of parameters derived from the JIP-test. Information in detail was shown in Table 1

**Table 1:** The variation of fluorescence transient parameters of Bermudagrass under shade

	A	B	C	D	E		Definitions
**Data extracted from the recorded fluorescence transient OJIP**							
**F**_**0**_	0.61b	0.64ab	0.67a	0.66a	0.67a		Fluorescence at time 20 µs after onset of actinic illumination
**F**_**m**_	2.28c	2.31bc	2.36b	2.48a	2.32bc		Maximal recorded fluorescence intensity, at the peak P of OJIP
**F**_**k**_	1.41b	1.45ab	1.50a	1.51a	1.25c		Fluorescence value at 300 µs
**F**_**j**_	1.63a	1.64a	1.67a	1.69a	1.44b		Fluorescence value at the J-step (2 ms) of OJIP
**F**_**i**_	2.00c	2.08b	2.12b	2.21a	1.97c		Fluorescence value at the I-step (30 ms) of OJIP
**Fluorescence parameters derived from the extracted data**							
**Area**	49.35b	48.09b	49.15b	52.59b	58.74a		Total complementary area between the fluorescence induction curve and F = F_m_
**F**_**v**_	1.66b	1.67b	1.68b	1.82a	1.65b		Maximal variable fluorescence
**V**_**k**_	0.48a	0.49a	0.49a	0.47a	0.35b		Relative variable fluorescence at k step
**V**_**j**_	0.61a	0.60a	0.60a	0.55b	0.46c		Relative variable fluorescence at J step
**V**_**i**_	0.84b	0.86a	0.86ab	0.85ab	0.79c		Relative variable fluorescence at I step
**M**_**0**_	1.91a	1.95a	1.97a	1.87a	1.39b		Approximated initial slope (in ms − 1) of the fluorescence transient
**S**_**m**_	29.70b	28.76b	29.18b	28.96b	35.69a		Normalized total complementary area above the O-J-I-P transient
**Ss**	0.32ab	0.31b	0.30b	0.30b	0.33a		Normalized total complementary area corresponding only to the O-J phase
**N**	93.06b	93.39b	95.82b	98.02b	106.59a		Turnover number: number of Q_A_ reduction events between time 0 and tF_m_
**Quantum yields and efficiencies**							
**φP**_**0**_	0.73a	0.72a	0.71a	0.73a	0.71a		Maximum quantum yield of primary photochemistry (at t = 0)
**Ψ**_**0**_	0.39c	0.40c	0.40c	0.45b	0.54a		Efficiency/probability that an electron moves further than Q_A_-
**φE**_**0**_	0.28c	0.29c	0.28c	0.33b	0.38a		Quantum yield of electron transport (at t = 0)
**φD**_**0**_	0.27a	0.48a	0.29a	0.27a	0.27a		Quantum yield (at t = 0) of energy dissipation (at t = 0)
**φR**_**0**_	0.12b	0.10c	0.10c	0.11bc	0.15a		Quantum yield for reduction of end electron acceptors at the PSI acceptor side
**δR**_**0**_	0.42a	0.34b	0.36b	0.34b	0.39a		Efficiency/probability with which an electron from the intersystem electron carriers moves to reduce end electron acceptors at the PSI acceptor side (RE)
**γ RC**	0.19a	0.18a	0.18a	0.13b	0.19a		Probability that a PSII Chl molecule functions as RC
**RC/ABS**	0.22a	0.22a	0.22a	0.22a	0.24a		QA-reducing RCs per PSII antenna Chl (reciprocal of ABS/RC)
**Specific energy fluxes (per Q**_**A**_ **-reducing PSII reaction center/RC)**							
**ABS/RC**	4.29a	4.49a	4.60a	4.64a	4.22a		Absorption flux (of antenna Chls) per RC (at t = 0)
**TR**_**0**_ **/RC**	3.13ab	3.25ab	3.28ab	3.39a	2.99b		Trapping flux (leading to Q_A_ reduction) per RC (at t = 0)
**ET**_**0**_ **/RC**	1.22b	1.30b	1.31b	1.52a	1.61a		Electron transport flux (further than Q_A_−) per RC (at t = 0)
**DI**_**0**_ **/RC**	1.16a	1.24a	1.32a	1.25a	1.22a		Dissipated energy flux per RC (at t = 0)
**Phenomenological energy fluxes (per excited cross section/CS)**							
**RC/Cs**_**0**_	0.14a	0.19a	0.15a	0.14a	0.16a		Density of RCs (QA-reducing PSII reaction centers) (at t = 0)
**ABS/Cs**_**0**_	0.61a	0.85a	0.67a	0.66a	0.67a		Absorption flux per CS, approximated by F_0_ (at t = 0)
**TR0/Cs**_**0**_	0.45a	0.61a	0.48a	0.49a	0.48a		Trapped energy flux per CS (at t = 0)
**ET0/Cs**_**0**_	0.18a	0.24a	0.19a	0.22a	0.26a		Electron transport flux per CS (at t = 0)
**DI0/Cs**_**0**_	0.17a	0.91a	0.19a	0.18a	0.20a		Dissipated energy flux per CS (at t = 0)
**Performance indexes**							
**PI**_**ABS**_	0.41c	0.39c	0.36c	0.49b	0.67a		Performance index (potential) for energy conservation from exciton to the reduction of intersystem electron acceptors
**PI**_**Total**_	0.30b	0.20b	0.20b	0.25b	0.44a		Performance index (potential) for energy conservation from exciton to the reduction of PSI end acceptors
**PI**_**CS**_	0.25b	0.33ab	0.24b	0.32ab	0.45a		Performance index on cross section basis

Note: The mean values from five biological replicates were listed in Table 1, followed by the letters showing statistical differences. ANOVA with Student-Newman-Keuls (*P* < 0.05) was conducted in present research

Increased shading appeared to remodel chlorophyll a fluorescence (O-J-I-P) transient curve (Fig. 2A). The variable fluorescence (F_V_) elevated from group A to D and plummeted in group E (Fig. 2B; Table 1). We separated the curve into two segments, O-J and J-P phase, based on the count of Q_A_^−^ reductions. The O-J phase witnessed a noticeable increase in the initial fluorescence (F_0_) from group A to C, seemingly indicating an enhancement of light-harvesting capacity, given the parallel increase in LHC transcriptional levels and photosynthetic pigments as shown in Fig. 1A. As J-P phase variation surpassed that of the O-J phase (Fig. 2A), we focused our analysis on electron transfer parameters beyond Q_A_^−^. Electron transport flux further than Q_A_^−^ per RC (ET_0_/RC) visibly surged in group D and E treatment compared to the control.

φE_0_ (efficiency/probability that an electron moves further than Q_A_^−^), Ψ0 (quantum yield of electron transport), S_m_ and N (the pool size of the electron acceptor beyond Q_A_^−^), and PI_ABS_ (Performance index for energy conservation from exciton to the reduction of intersystem electron acceptors) demonstrated a comparable trend (Fig. 3A, B; Table 1). These findings suggest an enhancement in electron transport under intense shade stress. Furthermore, the transcription levels of *PGR5/PGR5L*, a cyclic electron transport key component, were triggered by shade signal in groups A to C (Fig. 2D). Concurrently, parameters φP_0_,

ABS/RC and TR_0_/RC remained unaffected by shade, implying that the absorption and trapping of light energy per RC remains stable despite shading.

### The accumulation and transport of carbohydrates were impeded under shade

**Fig. 4:**
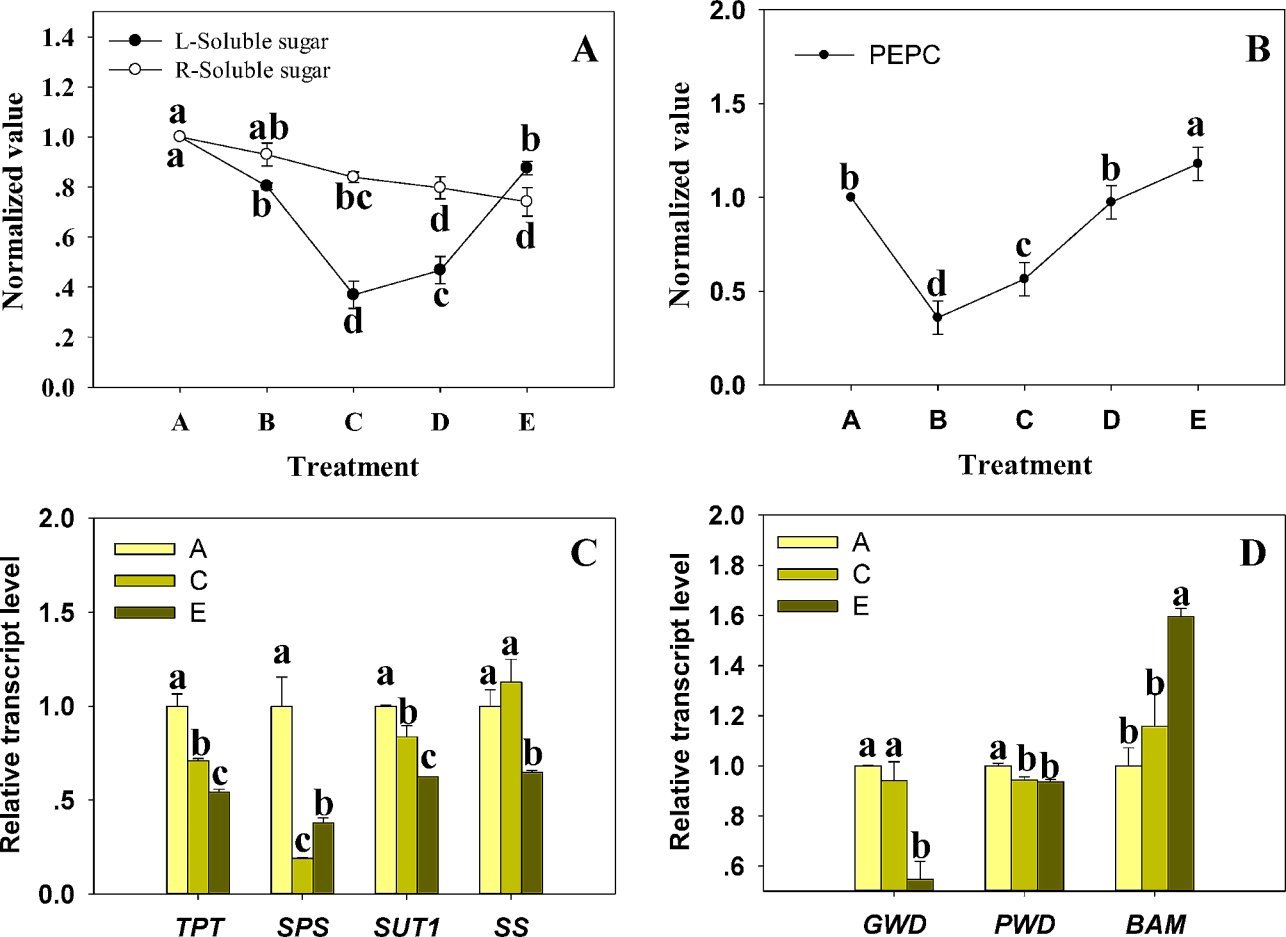
The carbon metabolism of Bermudagrass under shade. (**A**) Soluble sugar content in leaves (L) and roots (R); (**B**) Key enzymes (PEPC, Phosphoenolpyruvate carboxylase) activities associated with carbon assimilation in leaves; Transcript levels of genes connected with sucrose metabolism (**C**) and starch decomposition (**D**) in leaves. Protein names and functions of the corresponding genes: TPT (Triose phosphate/phosphate translocator, providing sucrose synthesis precursors), SPS (Sucrose-phosphate synthase, key rate-limiting enzyme in sucrose biosynthesis), SUT1 (Sucrose transport protein, transporting sucrose to the sieve element-companion cell for phloem loading), SS (Sucrose synthase, participating in the breakdown of sucrose), GWD (Alpha-glucan water dikinase, required for starch degradation), PWD (Phosphoglucan-water dikinase, required for starch degradation), BAM (Beta-amylase, required for starch degradation). Group A was considered as 1. A one-way ANOVA with the Student-Newman-Keuls test (*P* < 0.05) was carried out between various points on the same line or between different bars in the same set of histograms. Columns or lines marking the letters were ascribed to signify Mean ± SD and significant discrepancies. Five biological replicates were utilized

Our analysis showed that the content of soluble sugar in leaves displayed a U-shaped pattern, reaching its lowest point in the C group as the shading increased, as shown in Fig. 4A. Conversely, root sugar content linearly diminished with each subsequent group (Fig. 4A). Following this, we investigated the enzyme activity and transcription levels of key components entailed in the synthesis, transport, and decomposition of carbohydrates like sucrose and starch to gain insight into the reasoning behind this. The in vitro enzyme activity tests showed that the activity of PEPC (Phosphoenolpyruvate Carboxylase) was decreased by shade (Fig. 4B). However, it recovered with increasing degree of shading (Fig. 4B). Moreover, an observed down-regulation was witnessed with the transcription level of TPT, SPS, SUT1, SS with increasing shade levels (Fig. 4C). This obstruction in leaf sucrose metabolism could very likely have prompted the observed decrease in soluble root sugar, given that the transport of carbohydrates in plants primarily occurs as sucrose. Additionally, a unique up-regulation was discovered in Beta amylose (BAM) transcription levels (Fig. 4D). This may well have been a corollary of the escalating soluble sugar content in groups D and E. However, the degradation of starch did not counterbalance the continuous decline in the soluble sugar content in the roots that came with increasing shade, as observed in Fig. 4D.

### The nitrogen assimilation adjustments of plant in shade surrounding

**Fig. 5:**
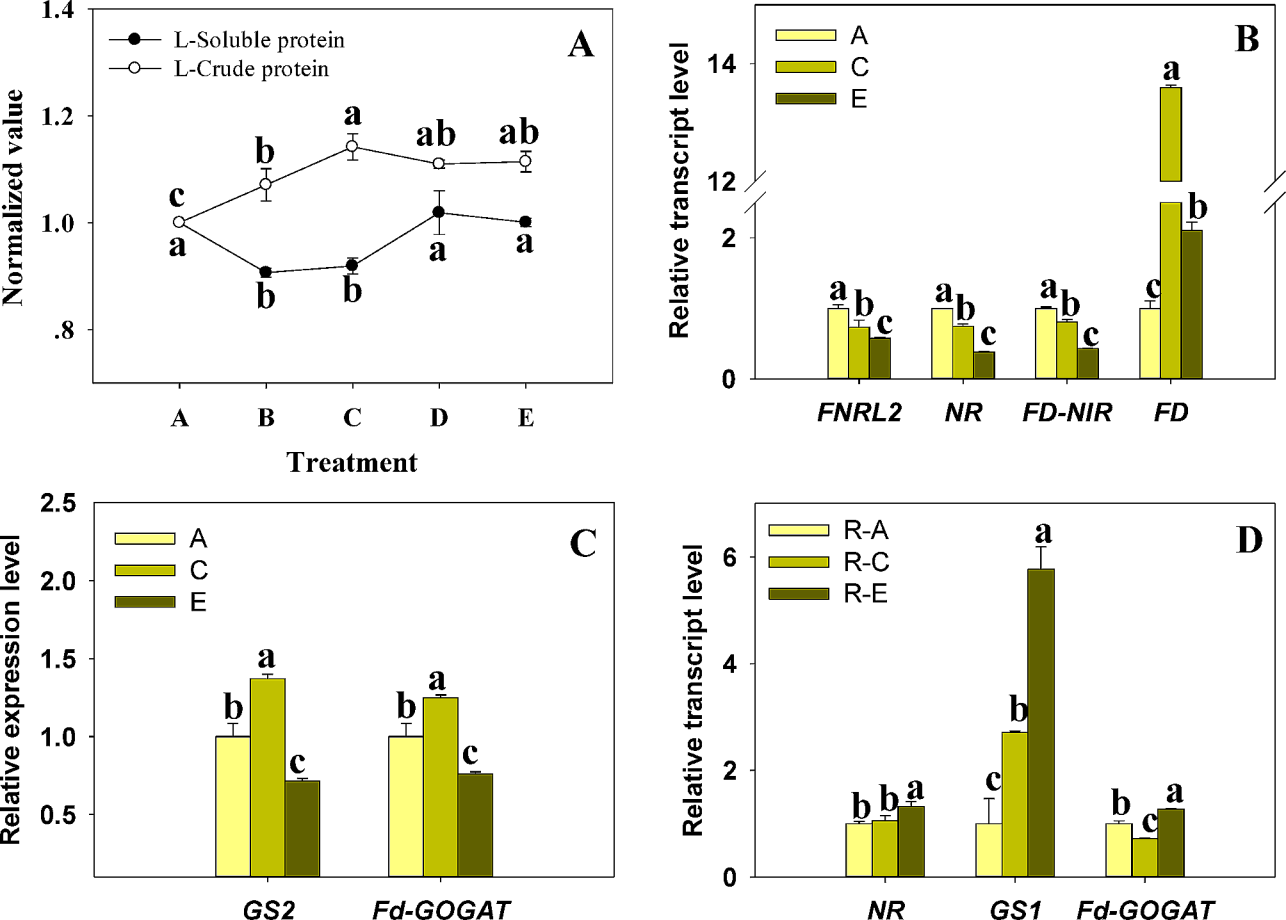
The nitrogen metabolisms in Bermudagrass under shade. (**A**) Soluble protein and crude protein content in leaf; (**B, C, D**) Transcriptional levels of genes related to nitrate reduction and ammonia assimilation. In the legend, “A” and “R-A” respectively denote leaves and roots of group A, and a similar nomenclature applies to groups C and E. Protein names of the corresponding genes are: FNRL2 (Ferredoxin-NADP reductase, leaf isozyme 2), NR (nitrate reductase), FD-NIR (Ferredoxin-nitrite reductase), FD (Ferredoxin), GS1 (Glutamine synthetase, cytosolic isozyme), GS2 (Glutamine synthetase, leaf isozyme, chloroplastic), Fd-GOGAT (glutamine oxoglutarate aminotransferase). The statistical methods and difference labeling mirror those of Fig. 4, with group A defined as 1

Carbon and nitrogen metabolism in plants was inherently coupled and competitive, which form the material basis of crop yield and quality respectively. Observations uncovered increased crude protein content (an essential measure of forage quality) per leaf dry weight within mild shade (Fig. 5A). However, soluble protein content appeared to decline in low shade and then subsequently rebound as shading intensified (Fig. 5A). This trend indicates a shift in the allocation of nitrogen within Bermudagrass throughout different shading conditions. Organic nitrogen stems from the transformation of inorganic nitrogen (NO_3_^−^, NH_4_^+^) through nitrogen assimilation. Our current study examined the transcriptional level of enzymes involved in this process. As the shading deepened, the transcript levels of *FNRL2*, *NR*, and *NIR* exhibited a gradual decrease (Fig. 5B). This suggests that shading might inhibit the conversion of nitrate to ammonium in leaves. The expression level of *FD* increased in light shade and decreased in heavy shade. Interestingly, *GS2* and *Fd-GOGAT* transcripts mirrored similar patterns as the level of FD (Fig. 5B, C). This could be because ferredoxin delivers the reducing capacity for the latter two. Simultaneously, shading resulted in an elevation of *NR* and *GS1* levels within the root (Fig. 5D). Taken together, our findings propose that both the diminished reduction force and deficiency in carbon assimilation caused by shedding light intensity might contribute to the alteration of nitrogen assimilation in shaded conditions. Accordingly, roots shoulder more nitrogen assimilation responsibilities than chloroplasts under shade.

### The contrasting antioxidant enzyme activities of leaves and roots in response to shading

**Fig. 6:**
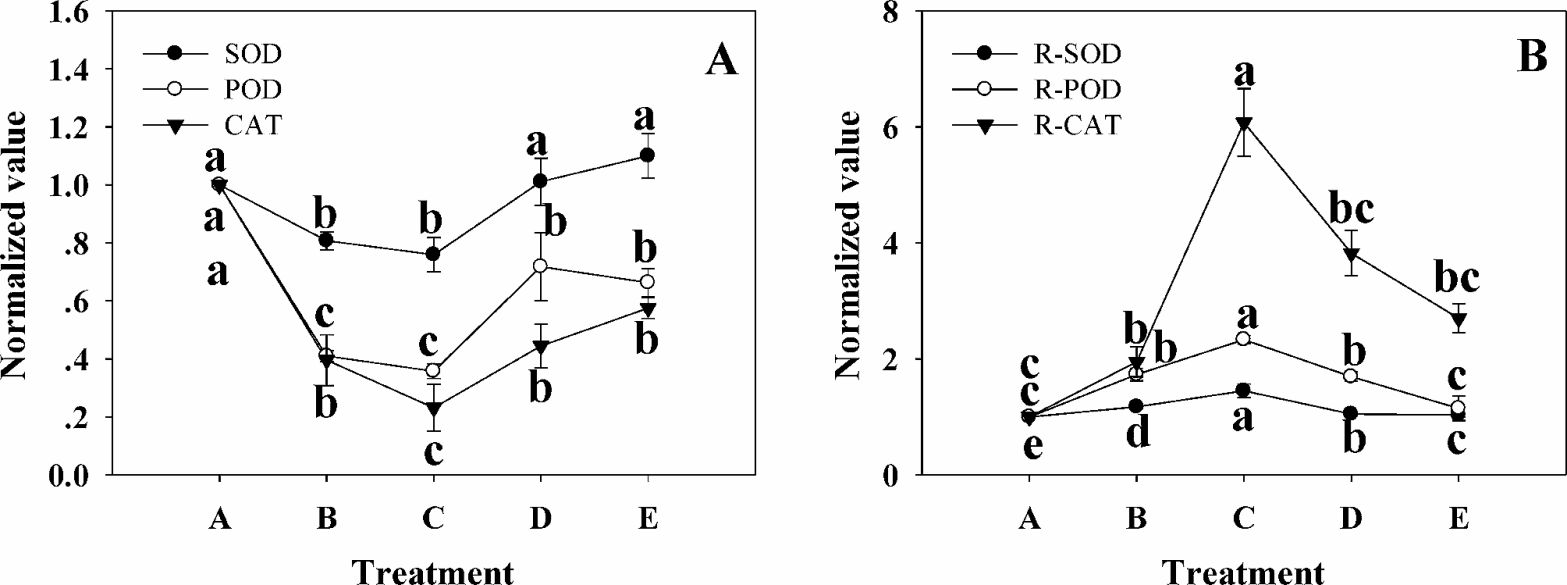
Antioxidant enzyme activities (**A, B**) in roots and leaves under shading. In the legend, “SOD” and “R-SOD” refer to Superoxide dismutase activities in leaves and roots, respectively. It applies equally to POD (Peroxisome) and CAT (Catalase). A group was defined as 1. One-way ANOVA with Student-Newman-Keuls (*P* < 0.05) was performed between different points on the same line. Lines marking the letters indicated Mean ± SD and statistically significant differences. Five biological replicates were utilized for the experiments

It was found that enzymatic activities changed in direct opposition under different light intensities in leaves compared to roots (Fig. 6A, B). Specifically, the SOD, POD, and CAT enzyme activities in leaves presented a U-shaped curve with light intensity reductions and estimated to the lowest in group C (Fig. 6A). Even though enzyme activities appeared to recover in groups D and E, they remained below their initial levels. By contrast, SOD, POD, and CAT enzyme activities in roots followed a parabolic trajectory with increased shading degree, peaking in group C (Fig. 6B).

## Discussion

It is widely accepted that shade-tolerant plants display a lower chlorophyll a/b ratio but a higher PSII/PSI ratio than plants in sunny environments [15]. Teramoto et al. [52] and Wu et al. [53] indicated that the transcription levels of genes encoding LHCII proteins (including LHCB2), in *Chlamydomonas reinhardtii* or *Camellia oleifera*, increased in low-light environments in low light (50 µmol m^− 2^ s^− 1^) compared to medium light (200 µmol m^− 2^ s^− 1^). Notably, Chlorophyll b is instrumental in controlling the antenna size of the photosynthetic apparatus and ensuring LHCII stability [54–56]. Our study demonstrated that the chlorophyll b/aratio and the level of LHC transcripts initially rise and subsequently decline with diminishing light intensity (Fig. 1A, C). We hypothesize that the increasing LHC may correspond with the decrease in chla/chlb observed from group E to B. Furthermore, we observed a steady augmentation in carotenoid content as the shading grew deeper (Fig. 1D). Previous research suggested that PIFs suppress *HEMA* expression [57] and *PSY* [58]. However, PAR1 prevents PIF1 from inhibiting *PSY1* expression in shaded conditions [59]. This alteration in *PSY* transcription levels may explain the increased carotenoid content recorded. Shading creates a low-light environment that substantially changes the content and composition of photosynthetic pigments, thereby profoundly affecting the input of light energy in photosynthesis. Based on these observations, we tentatively suggest that bermudagrass resistance to progressively deeper shade can transition from an active adaptation to passive tolerance.

State transitions have been suggested as a way to maximize light harvesting efficiency at low light intensities [60, 61]. A high proportion of far-red light under shade conditions dephosphorylates LHCII and shifts the photosynthetic apparatus to state 1 [62], where which LHCII detaches from PSI and rebinds to PSII. This process triggers an increase in overall chlorophyll fluorescence (ChlF) yield in “state 1” and a decrease in “state 2”, as the ChlF yield of PSI was much lower than that of PSII at room temperature [63]. In our study, we noted that F_0_, F_m_ and F_V_ increase with deeper shading from group A to D (Figs. 2A, B and C and 3A; Table 1), which aligned with the previous reports.

Under low light conditions, plants aim to maximize light harvesting which consequently makes them vulnerable to slight surges in light intensity. Non-photochemical quenching (NPQ) serves as a protective buffer during the activation of photosynthesis in low light until CO_2_ assimilation is adequately stimulated [64]. The induction of NPQ is partially due to the swift initiation of the cyclic electron flow (CEF), a process largely governed by proton gradient regulation 5 (PGR5) and the PGR5-like photosynthetic phenotype 1 (PGRL1) [44]. The intermolecular disulfide bonds of PGRL1, formed in the dark, are reduced with the commencement of low light, accompanied by a brief surge in NPQ [65]. The transcription levels of PGR5 and PGR5LA, PGR5LB, as depicted in Fig. 2D, exhibit an upregulation under shade in Group C. On the other hand, linear electron transfer generates a static ATP to NADPH ratio of approximately 1.3–1.5, which is inadequate to meet the energy demands of C4 photosynthesis. This ATP demand rises proportionally with the quantity of CO_2_ that escapes from the bundle sheath back to the mesophyll cells in shaded conditions. To counterbalance this additional ATP demand in PEP regeneration, C4 plants initiate cyclic electron flow (CET) around photosystem I (PSI) [66]. Following this reasoning, bermudagrass could potentially respond timely to the excessive energy induced by light fluctuations and uncoupling of carbon assimilation in shaded conditions by fortifying the PGR5-facilitated NPQ pathway. Simultaneously, this could account for the diversion of photosynthetic electron transfer over Q_A_^−^ under shade, leading to a significant increment in ET_0_/RC, φE_0_, and Ψ_0_ in groups B to E, relative to the control, as observed in this study (Fig. 3A, B; Table 1).

The enzyme NADPH: protochlorophyllide (Pchlide) oxidoreductase (POR) is known to catalyze the transformation of Pchlide into chlorophyllide under illumination and ultimately into chlorophyll. In our research, we reported that the transcript levels of *PORA* showed an increase corresponding with progressive shading (Fig. 1B). This observation is consistent with prior studies, wherein PORA was found to accumulate at protein levels during skotomorphogenesis [67]. We speculated that this phenomenon might be associated with the ability of the functional POR: Pchlide complex to mitigate ^1^O_2_ production risk during greening, thereby preventing photobleaching post-illumination [68]. Functionally, this seems reminiscent of the defense mechanism provided by PGR5 against sudden photo-oxidative damage.

C4 plant species exhibit distinct morpho-anatomical and biochemical variances in carbon fixation mechanisms compared to their C3 counterparts [69]. The C4 photosynthesis involves a process of carbon fixation that utilizes a CO_2_-concentrating mechanism (CCM), operational in the mesophyll cells (MS) and the bundle sheath cells (BSC). Initially, the enzyme phosphoenolpyruvate carboxylase (PEPC) catalyses the formation of oxaloacetate (OAA) from CO_2_ and PEP, which is subsequently reduced to malic acid. This malic acid permeates into the BSC, leading to the decarboxylation of CO_2_, which then enters the Calvin cycle. Consequently, CCM elevates the concentration of CO_2_ around Rubisco, substantially decreasing Rubisco’s oxygenase activity. Under conventional conditions, the efficiency of carbon assimilation in C4 plants typically surpasses that of C3 plants. This is attributed to the higher energy demands of photorespiration in C3 plants when compared to the operation of CCM in C4 plants [70].

Contrarily, under shade or fluctuating light conditions, C4 species exhibit reduced photosynthetic capacity and phenotypic plasticity in comparison to C3 species. This can mainly be attributed to the requirement of C4 species for more enzymatic steps than C3 species to be activated by light, which is important for promoting the metabolite gradient between MC and BSC [71]. Consequently, this leads to lesser CO_2_ assimilation (or CO_2_ leakage) [72], and an inhibited ability to use inconsistent light effectively [50]. Depending on the primary decarboxylase in the BSC, C4 plants are classified into three biochemical isoforms, which are NAD-ME, NADP-ME, and PCK. Among them, the NAD-ME plants exhibit the least adaptation to low light [51]. Past research has demonstrated that shadow diminishes PEPC activity and initial Rubisco activity in all C4 grass subtypes. However, the PEPC inactivation in NAD-ME experiences minimum reduction due to shading [51, 73, 74]. The PEPC enzyme undergoes activation via phosphorylation. This process is facilitated by calcium-independent serine/threonine protein kinase (PEPC-PK), whose transcription and protein synthesis are reliant upon light [75]. This phenomenon serves as a pivotal explanation for the decline in PEPC activity under shaded conditions (Fig. 4B). In our investigation, we additionally observed a resurgence in PEPC activity with the deepening of shade (Fig. 4B), and we propose several potential rationales for this observation. Phosphorylated PEPC exhibits greater resistance to proteolysis in comparison to its dephosphorylated counterpart. Furthermore, phosphorylation engenders specific docking sites for protein-protein interactions, and the involvement of 14-3-3 protein may induce conformational alterations or influence the interactions of target molecules upon binding to distinct phosphorylation sites on various target proteins [75]. We posit that 14-3-3 protein might modulate protein stability through its engagement in protein interactions in shade. Moreover, previous research has demonstrated that both 14-3-3 protein and PEPC serve as binding proteins for phosphatidic acid (PA) [76], an inhibitor of PEPC activity. Thus, 14-3-3 protein could potentially restore PEPC activity in shaded environments by competitively binding to PA.

Sugar serves not only as an energy source and vital component of structural material in plants but also as a signal that regulates the expression of genes and enzymatic activities [77]. The Sucrose Non-Fermentation-Associated Kinase (SnRK1) is part of a protein kinase family pivotal to energy and metabolic homeostasis [78]. Past research indicated that SnRK1 phosphorylates and inactivates Sucrose Phosphate Synthase (SPS) and Nitrate Reductase (NR) [79], and is essential for the transcription of genes such as Sucrose Synthase (SS) and α-Amylase (α-AMY) [80, 81]. In the HXK1-dependent sugar signaling pathway, HXK1 plays a role in the transcriptional repression of genes associated with photosynthesis (e.g., Rubisco and LHC) due to the presence of glucose [14, 82]. This study observed a drop in LHC and SS transcript levels in shaded Bermudagrass, correlating with an increase in soluble sugar content (group C to E) within the leaves (Figs. 1D and 4C). This mirrors patterns noted in previous research. Light signaling also plays a role in carbon metabolism, as prior studies showed that promoter activities of OsSPS1 and OsSPS11 are not governed by sucrose levels but rather by light levels and biological clock [83]. Moreover, in the Cryptochrome 1 A (CRY1A)-mediated blue light signaling pathway, HY5 binds directly to the promoters of starch-degradation-related genes, such as PWD, BAM1, BAM3, and BAM8, triggering starch degradation [84]. This specific regulatory pattern was reaffirmed in the current study that showed reduced *SPS* and elevated *BAM* transcript levels under low light conditions **(**Fig. 4C, D).

Optimization of plant performance in fluctuating environments is achieved by coordinating above-ground photosynthetic carbon fixation with root inorganic nitrogen uptake. The photosynthetic process plays a crucial role in providing the energy and carbon skeletons necessary for nitrogen assimilation in plants. However, the distribution of nitrogen within the photosystems significantly influences the efficiency of photosynthesis [22]. Plants have evolved mechanisms to optimize the allocation of nutrients, aiming to achieve a state of “functional balance.” Past research also shows increased allocation of nitrogen to components such as light harvesting components (LHC) and water-soluble protein under low light conditions [53]. Our findings support this, indicating an increase in soluble protein content under shade (Fig. 5A). An observed rise in crude protein content may be partially attributed to an increase in LHC. Additionally, due to Rubisco’s inherently low catalytic turnover rate and its tendency for competitive oxygenation reactions, photosynthesis in C3 plants is often limited by the capacity of Rubisco. Consequently, higher plants tend to accumulate substantial amounts of Rubisco, which requires a significant investment of nitrogen. Similarly, in C4 plants, carbon limitation can occur in the Rubisco of bundle sheath cells. Despite having lower concentrations of Rubisco compared to C3 plants, C4 plants still accumulate significant amounts of Rubisco. Insufficient levels of nitrogen may restrict the metabolic flux required for enzyme production in plants [85]. Generally, in C4 plants, the activity of Rubisco enzyme decreases rapidly following shading, as a mechanism to reduce the nitrogen investment in leaves. NAD-ME type plants, however, tend to allocate more nitrogen to Rubisco, in order to optimize photosynthetic efficiency under low light conditions [51]. This was supported by the results in the present study showed that more soluble proteins were up-regulated by deeper shading in group D and E (Fig. 5A). It is important to note that the activity of Rubisco activase, which is light-dependent, decreases under low light conditions. Therefore, although there is an increased input of nitrogen into Rubisco, this allocation may not effectively enhance Rubisco’s catalytic activity in carbon fixation, leading to nitrogen wastage. Notably, the decrease in Rubisco activity under shading occurs faster than that of PEPC, especially in the NAD-ME biochemical subtype of C4 plants [86]. In other words, the activity of PEPC under shading is relatively less sensitive to changes in nitrogen allocation compared to Rubisco. In the present study, we observed that the PEPC activity was reduced by shading in general, and recovered somewhat when N assimilation was inhibited by deepening shading (Figs. 4B and 5B), probably because PEPC activity was more stable in the limited N environment.

HY5, acting as a mobile signal from shoots to roots, moderates root growth and nitrate uptake response to light [87]. Indeed, light intensity triggers key enzymes of nitrogen metabolism such as NR, NIR, GS. In terms of transcriptional regulation, both NR and NIA2 (NIR) are regulated directly by HY5 [88, 89]. Our evidence indicates NR transcript down-regulation in leaves yet up-regulation in roots under shade conditions (Fig. 5B, D), suggesting HY5’s possible role in nitrogen fixation coordination between leaf and root. This implies roots assume some consequences of reduced leaf nitrogen metabolism under low light. Plants have multiple isozymes of the enzyme GS, which can be classified into GS2 (plastid-localized) and GS1 (cytoplasmic-localized). GS2, primarily expressed in leaves, catalyzes the re-assimilation of ammonia in photorespiration [90, 91]. In contrast, GS1 is typically detected only at low levels and often limited to the phloem, where it functions in non-photorespiratory ammonia assimilation [92]. In our study, distinct transcriptional profiles of GS were found in leaves and roots. While leaf GS2 transcripts were only up-regulated in group C (Fig. 5C), seeming to reflect the pattern of LHC, PGR5 expression, evidence of NR and GS1 transcripts increased in roots with shade intensification (Fig. 5D). This outcome may be due to energy overflow from carbon assimilation blockage under shaded conditions, leading to partial consumption by photorespiration, thereby inducing GS2. While the increased GS transcript levels in root were to match nitrogen assimilation levels.

Decoupling between light reaction and carbon fixation in photosynthesis under low light results in an excessive reduction in the photosynthetic electron transport chain and leads to the generation of Reactive Oxygen Species (ROS). Principal agents contributing to ROS production in plants include the photosynthetic electron transport chain, photorespiration, the respiratory electron transport chain, and NADPH oxidase situated in the plasma membrane [93]. This study demonstrates that leaves and roots exhibit markedly contrasting alterations in antioxidant enzyme activities under shaded conditions (Fig. 6A, B). It is suggested that the sources of ROS in root and leaf cells might vary due to their subcellular and functional heterogeneity. Previous studies have shown inconsistent results regarding antioxidant enzyme activities in leaves, contingent upon the shading duration and the species studied. A ten-day absence of light resulted in decreased CAT activity in Arabidopsis leaves, while low light led to increased activities of SOD, POD and CAT in soybean leaves [94]. Nonetheless, all the plant species studied were of the C3 variety. A speculated overall decrease in antioxidant capacity in NAD-ME plants is believed to indicate a greater capacity to transport reducing capacity to the Calvin cycle [66]. Insights from Bräutigam and Gowik’s study [95] explain why, to a certain extent, bermudagrass retains its ability to deliver a reductive power source to the Calvin cycle via photorespiration and cyclic electron flow when in light shade. According to the researchers, a sufficient flux from photorespiration is required for C4 photosynthesis to function as a CO_2_ pump, therefore channeling CO_2_ into the bundle sheath through glycine decarboxylase. However, as the shade intensifies and the level of antioxidants such as glutathione decreases [96], there becomes a greater requirement for antioxidant enzyme activity due to the accumulation of H_2_O_2,_ a by-product of photorespiration.” Ha et al. [97] demonstrated that, in order to detoxify reactive oxygen species (ROS), phyB stimulates the biosynthesis of abscisic acid (ABA) in shoots, transferring this signal to the roots which induces peroxidase activity. Similarly, HY5 instigates phloem loading of sucrose via direct activation of the SWEET11 and SWEET12 genes, thereby encouraging root development [88]. Moreover, the overexpression of SnRK1 increases the tomato’s salt stress tolerance by elevating activities of superoxide dismutase (SOD), peroxidase (POD), and catalase (CAT) [98] (Fig. 6A **B**). It is thus proposed that an increase in antioxidant enzyme activity in the roots when under shade is potentially linked to ROS accumulation, likely due to phyB inactivation or energy deficiency, and thus appears to coordinate stem and root growth.

To explore unclear mechanics of C4 plants’ response to shading, this study undertakes a comprehensive analysis of the physiological and biochemical characteristics exhibited by the C4 plant Bermudagrass under varying levels of shade. These findings have implications for breeding and expanding the application of shade-tolerant grasses. However, this study does have potential limitations; for instance, the effect of light intensity was not distinguished from the quality of light on Bermudagrass under shade, an area which requires further work to elucidate.

## Conclusion

In shaded environments, Bermuda grass demonstrates sophisticated regulation of both light harvesting and subsequent electron transfer processes. Moderate shading induces an increase in Chl b and LHC transcripts, while intense shading triggers the accumulation of Chl a, carotenoids; and electron transfer beyond Q_A_^−^ (ET_0_/RC, φE_0_, Ψ_0_). This investigation underscores the disparate impacts of shading on leaf and root physiology. Shading diminishes the transcript levels of *SPS* and *SUT1* while enhancing those of *BAM* in leaves, consequently altering soluble sugar concentrations between leaves and roots. Furthermore, shading diminishes the transcriptional activity of nitrogen assimilation genes (e.g., *NR*) and the enzyme activities of SOD, POD, and CAT in leaves, yet augments them in roots. Orchestration of sugar, light and ROS signals may account for these adjustments in photosynthetic acclimation.

## Methods

### Plant material

This study utilized the herbage-type cultivar, ‘Wrangler’ Bermuda grass, originating from the grass resource germplasm nursery at Ludong University. To mitigate the inter-individual variations of seeding, stolon from the parent plant was uniformly propagated. A seedling-raising tube (5 cm in diameter and 25 cm deep) filled with silver sand and regularly watered with half-strength Hoagland’s solution (1/2 HS) (200 ml per week) was employed as a culture system. A node of stolon, covered with wet sand, took root and developed into a complete seeding within a controlled greenhouse over a month. The growth conditions were maintained at 24/20°C for day/night, with 14/10 hours light/darkness, relative humidity of 40%, and natural light intensity of 500 µmol m^− 2^ s^− 1^(on average).

### Treatment

To emulate the environment of understory herbaceous species, a sunshade net (with a 50% shading coefficient) was utilized to create a light intensity gradient. Illuminance meters (DLY-1802, Delixi Co., Ltd, China) were installed to ensure the expected light intensity was achieved in these shaded environments. Consequently, Bermuda grass seedlings were divided into five groups as follows: (i) natural light (500 µmol m^− 2^ s^− 1^) (A); (ii) covered with one layer of sunshade net (250 µmol m^− 2^ s^− 1^) (B); (iii) covered with two layers of sunshade net (125 µmol m^− 2^ s^− 1^) (C); (iv) covered with three layers of sunshade net (62.5 µmol m^− 2^ s^− 1^) (D); (iv) covered with four layers of sunshade net (31.25 µmol m^− 2^ s^− 1^) (E). Shade conditions persisted for a week. The illumination intensity was separately recorded as in A, B, C, D, E. Each treatment comprised five duplications (tubes).

### Photosynthetic pigment examination

A total of 0.1 g of fresh leaves were collected and immersed in 5 ml of dimethyl sulfoxide maintained at 4 °C. Following a 24-hour incubation within dark, ultraviolet spectrophotometer, model TU-1901 (Persee General Instrument Co. Beijing), was utilized to measure the absorbance of the extract at wavelengths of 645 nm, 663 nm, and 440 nm. Using the method detailed by Li et al. [99], the pigment content was subsequently calculated.

### Chlorophyll a fluorescence transient and the JIP-Test

To investigate the organization and performance of the PSII in shade-grown bermudagrass, chlorophyll a fluorescence transient was obtained using a Pulse-Amplitude-Modulated (PAM) Chlorophyll Fluorometer model PAM2500 (Heinz Walz GmbH), thus yielding multiphase rise curves (O-J-I-P). Blade samples were subjected to dark adaptation for 30 min prior to the onset of 2s duration red saturated pulsed light (650 nm, 3500mmolm^− 2^s^− 1^). Vertical irradiation of the leaf surface was achieved with the aid of a leaf clip and optical fiber. Seedlings were routinely maintained under shade conditions with the exception of those undergoing measurement.

The O-J phase was further broken down into W_k_ = (Ft − Fo) / (F_K_−Fo) and ΔW_K_=W_K_ treatment -W_K_ ref (“ref” is for natural light in A group) in order to provide more detailed insights into the primary photochemical reaction process. In ΔW_K_ curve, the L-band (occurring roughly at 150 µs) suggested energetic connectivity among the components of PS II RC. In ΔW_K_ curve, a positive L-band indicated an increase in energetic connectivity in the treatment group, whereas a negative L-band indicated decreased connectivity.

Based on the principles of the “energy flow” model, the JIP-Test transforms the efficiency of light energy conversion and transfer into a numeric value, as per Strasser et al. [100, 101]. Specific fluorescence signals recorded at various time points (0.2 ms, 2 ms, 30 ms) provided considerable information about the absorption (ABS) and trapping (TR_0_) of light quantum, dissipation (DI_0_) in light-harvesting antenna, electron transport (ET_0_) though two optical systems, and reduction of end acceptors of PSI (RE_0_). The parameters involved were categorized into four groups: (1) basic measured and calculated values; (2) quantum yields and efficiencies; (3) specific energy fluxes; and (4) performance indices. Five replicates were maintained for each treatment.

### The assay of soluble sugar and protein

A 0.20 g portion of either freshly chopped leaves or roots was boiled in distilled water for 30 min. Following filtration, the volume of the filtrate was fixed, totaling 25 ml. 0.5 ml filtrate was used to detect soluble sugar content according to phenol method with sucrose as the standard [98].

In regards to the crude protein content, 0.20 g of dried leaves were digested with 10 ml H_2_SO_4_ via a graphite digester (SH220N, Hannon, China). The previous solution was then examined with an entirely automated Kjeldahl nitrogen determination instrument (K9860, Hannon, China), as the given instructions. Crude protein content (%) = nitrogen content × 6.25 × 100%. Soluble protein was determined by extracting the supernatant from 0.2 g of fresh leaves ground with liquid nitrogen using a pre-chilled phosphate buffer (pH = 7.8). The conditions for centrifugation were 12,000 × g at 4 °C for 20 min. 20 µl of the supernatant was mixed with coomassie brilliant blue (G-250) dye solution for 2 min. The light absorption at 595 nm was applied to estimate the soluble protein content [95], using bovine serum albumin as the protein standard.

### Enzyme activity analysis

The enzymes were extracted from 0.2 g of fresh leaves or roots through liquid nitrogen grinding and centrifugation. The activities of SOD (EC:1.15.1.1) and POD (EC:1.11.1.7) enzymes were analyzed as reported earlier with slight modifications. The 3 ml reaction mixture for SOD consisted of 50 mM phosphate buffer (pH 7.8), 195 mM Methionine, 20 mM riboflavin, 100 µM EDTA-Na2, 750 µM NBT, and 0.2 mL of the crude enzyme. The reaction system was illuminated for 30 min at 72 µmol m^− 2^ s^− 1^, and then, the absorbance at 560 nm was recorded. For POD, the reaction mixture included 20 mM guaiacol, 100 mM PBS (pH 6.0), 40 mM H_2_O_2_, and 0.2 mL of enzyme extract; its mean change in absorbance per minute was measured at 470 nm. The CAT (EC:1.11.1.6) reaction process involved 0.1 ml of the crude enzyme solution mixed with 1.9 ml of the 50 mmol/L phosphate buffer (pH 7.4) and 1 ml of 45 mmol/L H_2_O_2_. After sufficient mixing, the absorbance at 240 nm was measured using a spectrophotometer (TU-1901, Persee general instrument Co. Beijing.) The reaction system using distilled water instead of the crude enzyme served as a control. Measurements were taken at 1-minute intervals for 3 min, and a decrease of OD240 by 0.01 in 1 min was defined as one unit (U) of enzyme activity.

The enzymatic activities of PEPCase (Phosphoenolpyruvate carboxylase, EC:4.1.1.31) was determined as follows. The crude enzyme was derived from 0.2 g of fresh leaves using an extraction buffer that comprised 0.1 M Tricine-HCl (pH 8.4), 10 mM MgCl_2_, 1 mM EDTA, 7 mM β-mercaptoethanol, 5% glycerol (v/v), and 1% PVP, and then centrifuged at 15,000 × g, at 4 °C, for 10 min. A 2.5 ml reaction system was developed for gauging PEPCase activity. This involved 100 mmol Tris-HCl (pH 9.2), 10 mmol MgCl2, 10 mmol NaHCO3, 0.16 mmol NADH, 0.5 mmol PEP, malate dehydrogenase (15 U), and 0.5 ml of the crude enzyme. After maintaining a constant water bath at 28 °C for 10 min, the reaction was activated by PEP. Eventually, the activity of PEPCase was assessed via the decreasing rate of NADH at 340 nm.

### RNA isolation and quantitative real-time PCR

The extraction of mRNA was undertaken by applying the Plant Total RNA Purification Kit’s instructions (Gmbiolab. Co., Ltd, Taiwan) to 0.1 g of fresh leaf, which was later reverse transcribed to cDNA utilizing the Hifair™ II 1st Strand cDNA Synthesis Kit (YEASEN, Shanghai, China). The gene sequences utilized in this study were obtained from transcriptome sequencing data, and the primer sequences, designed by the Oligo7 software, are provided in Additional File 1.

Each reaction system, consisting of 2 µl cDNA template, 10 µl of SYBR Green master mix with low Rox (Yeasen, China), 0.5 µl of forward primers, 0.5 µl of reverse primers, and 7 µl of nuclease-free water, totalled 20 µl. The ABI Quantstudio 6 Flex real-time PCR system (Applied Biosystems, Foster City, CA) was employed to operate the qRT-PCR protocol, which included conducting a melting curves inspection at the end of each reaction. The *ACTIN* gene served as the reference, and five replications were made for each reaction.

### Statistical analysis

Each assay was carried out at least five times independently. ANOVA with Student-Newman-Keuls was used to determine the significance of the differences, with a P-value of less than 0.05 considered statistically significant. The dots or bars in the graph represent the mean plus or minus the standard deviation. The levels of significance were represented by a series of letters (a, b, c, d).

### Electronic supplementary material

Below is the link to the electronic supplementary material.

Supplementary Material 1

Supplementary Material 2

## Data Availability

Data sharing is not applicable to this article as all new created data is already contained within this article. RNA sequences of genes involving in manuscript are retrieved from the National Center for Biotechnology Information (NCBI) Sequence Read Archive (SRA) database (accession number: PRJNA645038) and listed in Additional file 2.
