# Supplementary material for: A comprehensive assessment of photosynthetic acclimation to shade in C4 grass (Cynodon dactylon (L.) Pers.)
Source: BMC Plant Biol. 2024 Jun 21;24:591. doi: 10.1186/s12870-024-05242-x (PMC11191358; doi:10.1186/s12870-024-05242-x)
Supplement: Supplementary file 1 — Supplementary Material 1 [file 12870_2024_5242_MOESM1_ESM.docx]

**The primer for qRT-PCR**

| **Gene** |  | **Primer sequence (5 '-3')** |
| --- | --- | --- |
| *HEMA* | F | GCAGTGGGCCGTGGAGAAGC |
|  | R | AGGGACTCGCTGGAGACCGTT |
| *PORA* | F | TGCTTAAATCATCGGCACCAAGTCGGA |
|  | R | TCTCGCCAATTCTCTCGTAAACAGGCA |
| *PSY* | F | TCTCCGGACGGCGATTCCCA |
|  | R | CCTCTGTTGGGCCGCTCTCC |
| *PDS* | F | CCGAGCAGAGGGTGCACGAG |
|  | R | CGATCGAACGCCGCCTCCAG |
| *LHCB2* | F | AGCGGTGTCCCACCCGTAGT |
|  | R | TTCCTCGGCCAGGCTCTCGT |
| *LHCA2* | F | CCCGGCGACTTCGGCTTTGA |
|  | R | GCCGGCGGTGTACCAGAAGG |
| *PGR5L1A* | F | TCGATGAGCTGACTGGCTTTGAGGT |
|  | R | GGTTACCACTTGCGCCGTAACCA |
| *PGR5L1B* | F | TGTTCCTGCTCAATGTTCCAGCGACT |
|  | R | CCGAAGGGCTCCGGAAACTGG |
| *PGR5* | F | CTCGCAGGCGCCACTGTTCT |
|  | R | CACGCCCAGGGCATTCCCAA |
| *TPT* | F | CGAGGCCACCTTGCCTCTCGAT |
|  | R | GGCGAGCTGCGAGAGTTTCGG |
| *SPS* | F | TCACCTGACCACCCGTGTCTGAA |
|  | R | TGCTGGCGATCCATCTGTTGCC |
| *SUT1* | F | CCATGGTCCTCGTTGGCATGCAG |
|  | R | GGGCCATGCCGACCTGACAA |
| *SS* | F | TTGGAGTCCACGCCCACCCT |
|  | R | AGGAACTGCTCCGCCTCAGGA |
| *GWD* | F | GCCTTCCACGCTTGCTCCCA |
|  | R | ACCTGATAGCTCCTGTCAGTCTGGTCA |
| *PWD* | F | CTGCGGCCTGCTCTGCACTA |
|  | R | CGGCTTGCTGCCGTTGGACA |
| *BAM* | F | GGTCGGGTCGCCGGAACTAC |
|  | R | CGCATGAAGTCGCCGTAGCACT |
| *FNRL2* | F | AGACAGGCGAGGAGGTGCCA |
|  | R | CGGTAGGGCGCAATGCCTGT |
| *NR* | F | TTCCACCATCGCGCTTGCCA |
|  | R | ATGCAGAGCTTGCCGCCGAT |
| *FD-NIR* | F | GGCGAGCGTGATCGATGCCT |
|  | R | GTCGATGCCGGCAAGTGGGT |
| *FD* | F | GAGGGCGAGGTGGAGCTGGA |
|  | R | GCTGGCCGGAGACCACCTTC |
| *GS1* | F | AGGATGTAGCGGGCCACCCA |
|  | R | CGACGTCGTTGACGCCCACT |
| *GS2* | F | GCGTGCCAGGGCTGCAGAAA |
|  | R | AGGCCAGCCAAGAGGCCAGT |
| *FD-GOGAT* | F | CACGGTGCTCCATGCACCCA |
|  | R | CCGTGCAGGAGGTTGCGGAT |
