# Supplementary material for: A comprehensive assessment of photosynthetic acclimation to shade in C4 grass (Cynodon dactylon (L.) Pers.)
Source: BMC Plant Biol. 2024 Jun 21;24:591. doi: 10.1186/s12870-024-05242-x (PMC11191358; doi:10.1186/s12870-024-05242-x)
Supplement: Supplementary file 2 — Supplementary Material 2 [file 12870_2024_5242_MOESM2_ESM.docx]

**Transcript of genes involved in manuscript**

>Cluster-342212.196217 HEMA

TTTTTGGATGATAACAGATAAAGAGAAGAATATAAAATAATAATAACATATCAACAGGCTGAAAGTCAGAAACAGGAATA

CCATCAACAGTACAGGATCAACCATGCAGGATACTAGTAAGATAAGCAGTACTCTACAGACTATAAAGCACATCCCTAGG

ATATGGTTAGCCCATATGGTTATAGGTGAACTGGACACAAAAGGATGTGAACTCTTAAAAAAGTGTTAATGCTCAACAGT

TGTAAGTACAGATCCAACGTAAATGGCAGTACAAAGTATCACTACAAGTTTAAACGCACAATGATGCTCCAGAGGGCATC

AACTGAGACATTATGTTTATTTCTTCCAATAGCGTATTAAGAATGGGGCTCTTGCCACAGCACCTGCAGTAATGTACAAT

TCGCTGCTATCTGAACAGATTTAGAGGCTGCCATGGCGCCAATTTCACGATACAGTTGACGATCTCAGCGAGCAGGGGGA

TCAGTAAGCGAAGGATCAAAAAAATTGGAATCAGAATGTTTATATAGATATACAGGTAGAAAAATTGCTTCCTGGCCTCA

GTTCTGGGTCTTCTCCACCTTTGCTCTGATCTTCTGCTCGACAATTGCCTTCTCTGTATCGAGGCTGAACATTCTATTGA

GGGCGTGCATGTTCTCAATTGTCTCGTCAAGTGTGCGGCTGTCGCTACCATCGCACCTCAGGTGCTGCAGTGGGCCGTGG

AGAAGCTTGTTCACGATGCCAGTGCTGAGCTCCTCGATTGCTCTTCTCATTTTCTTGGTGAGAGCATCTTCACCAATCTT

CTGCAGGCACTTCTCTAGCTCTGAAGCTCTGATCCTGTCGGCATATGATCTCAACTTCTTGATGGTTGGAACGGTCTCCA

GCGAGTCCCTCCATGCCTCAAACCGTTTCAGCTCCTGGGTGATGATTGTTTGTGCCTCCATTGCCTTCCTAAGCCGGTCT

TCCTTGTTGGCCTCCACAACCTCTTTCAAGTCATCGACATTGTATACCCGTGCATGGTCAACTTCAGACACGCACGCACT

GACATTCCTTGGGACTGATATGTCAACAAAGAACCGAACCCCACCCATGGCATCAGAAAAAGAAGGAAGTGCCTCTGCGT

GCTCTTTCGTGAACAATGGGGTTTCAGATGCAGTGCTTGTGAACACAACATCAGCTTCTGCAGCAGCTTCGTACATCTCT

GATAGAGGCCTGTACACAATCTCAATATCTTTCATCTCCTCGCGGATGGCATCCACCCTTTCCACTGAACGGTTCACTAC

AACAACCTTCTTGCATCCTTTCGCAATCAGATGTTTGATCACTAATTTGCCCATTTTGCCAGCACCAATCAACAACATCC

TCGCTGATAAGCCTTCAGACTTTGGAAGCTTCATCAGGGCCAACTCAACTGCTGCCGAACTAACAGAAACAGCACCAGAA

GATATGTTAGTCTCGCAGCGGACACGCTTTCCAGCTGTGATTGCATCCTTGAACATCCTATCAATGTTCTTTCCCAGGCC

TCCACTGTTTTGGCCACTTCTCACAACTTGTTTAACTTGAGCAAGAATCTGTCCTTCACCCAGAACCAGAGAGTCGAGCC

CAGCTGATACCTCGAACAGATGACGTGTAGCATCACTATCACGCAACATGAACAGGTGCTCCCTAAGCTCAGATGCAGGT

ATACCACTTTTCTTTGACATCCAGTCTATTACTTCTCTGATACCACGATTCCATGATAGCGCCACCACATAAATTTCCAT

TCTATTGCAGGTACTAAGTACAGCAGCCTCTTCAATATGATTCAGACTGGTGAGTTCTGAAATAGCACGGGGCCATAGTT

CCTCCGCAACAGCAAGTTTTTCACGCATCTCCACTGGTGCTGTGTGTACACTGAGGCCAATCACAGCTATGCTGCTTCTT

TCCTTCATGTACCGGTCGGCG

>Cluster-351287.0 PORA

AGCCAGCATGGAATCGATTAGAGAGTTTGCTAAGAATTTCAAATCGAAATTTAATAGATTAGATATATTAATTAACAATG

CTGGTTTAACATTAAAATCGAGAATGGAGACAAAGGATGGCTTTGAAATGTGCTTTGGAACTATGCATTTAGGTCATTTT

CTATTAACTGGTTTGTTGATTGATTTGCTTAAATCATCGGCACCAAGTCGGATTGTTAATGTATCTTCACTAGCGCACTT

TGGGACAAGTCTTGATTTCGATGACTTGCAAATGAAGAAAGACTACGAGCGATTTAGAGTCTATAGACGTTGTAAGCTCG

CTAATTGCCTGTTTACGAGAGAATTGGCGAGAAAATTCGGAACGATCGGAATCACTTCTGTTAGTCTGCATCCAGGAGTA

GTAAATAGCGATATAAATCGAGAAATGGAAGCCAATAGAGAATGGATATGGATACTCAGCATGATATTTAGGCCATTCAT

GTGGATGTTGTTTAAGACTATTCGACAAGGAGCTCAAACAACTATTCATTGCGCCATCGATGACGATGTCCCTAACCATA

ATGGCGACTATTTTAGGGACTGTAATCCTGCGCGTTGTTCAGCTCAATCGAAAGATGAAAAGAGTGCTAGAAAGTTATGG

GAGATTAGTGAAGAGCTGACAAAAATAAACTATTAGCTTATATTTAACAACAATTTTTTATTTACTATTAAATTATTACT

ATTAAATCTAATCTAAAATAATAAAATAAACTCCGGCATTTAAAAAAAAACACAATTTAGCTATTCAGTTAGCCATGTTT

GCCAAAAGAGGATTTGACATCTTTGAACAAACTTGAAATCGAG

>Cluster-342212.187805 PSY

TTTACAGATTTGACTCATTTGTACTGTTGAATTTTGTAGAATTGATATCTCTGAGATATAGGGATGAGAATGTAAAACGT

TGGAGAAGCTGAGGTGAAGAGTGAAGAAGACGTTCGACTTGAACGTTCGTGCGTGGGGCACGCAGGCGAACACCTCGCCG

GGACAACCAGACCACTCGCTCACCTGGACTCCAGAAGCTACCAGCGGAAGGAATGTGCTGGTGCGCGGCCGTCGTCGCCG

GAGGCCGGCTGCCTCCGCCCGCCCTCCCCTACCGCCTCAACCGCCGCCGCCGCCGAGGGACTTCCATCAGGGCAGAGTCT

TCTCCGGACGGCGATTCCCAGCGGAAGAAGGTCGCCATCGCCGGCGCCGGTTGGGCCGGACTCGCCGCAGCGCACCATCT

CGTCAAACAAGGGTACGATGTCACGCTTCTCGGGGCGGAGAGCGGCCCAACAGAGGAGGTTGGTCTCAGAGGATTCTGGT

ATCCGTATCGCAACATTTTCTCTCTAGTCGACGAGCTGGGGATCTCTCCTTTCACAGGCTGGAATAAAGCAGCGTACTAT

TCTCCGCATGGCCTTTCAGTCGAATTTCCTATTTTTCACAACCAGCCCAGGCTTCCAGCTCCCTTTGGAGTCTTTGCATA

CCCGGAATTCCCTAATCTTCCTTTGTTGGATAGATTGACATCAATTGCTGTCATAGCTGCAGTAATTGACTTTGACAACA

CAGACACTGCTTGGAGAAAGTATGACGCTATGACTGCAAGAGAGCTTTTTAAGATGTACGGTTGCTCTCAAAGGCTCCAC

AAGGAAGTCTTTGAACCAGCTATTCAGGCTTCCCTGTTTGCTCCTGGCGAGCAATGTAGTGCTGCTGCAACACTGGGGAT

GCTTTATTATTATATGCTCTCTCATCAGGAAAACTGTGATTTTTTGCTGTGCCGTGCGGAGGTAGAAGAAAAGATTTTCT

CTCCCTGGCTGAAATCATTGGAGTTGAAAGGCTTAAAATTTGTTGCAAACAAAATTCCAACAAGTATGACTATAGATAAA

GATAGTGGATGCATCTCTTCAATTATGTGTGGTGAGGATGTCTATGAGGCAGATGCATTTGTTTCAGCCATGGGACTCTC

TCTTCTACAGTCCATCATTACTAACAGCTCATTTCTGCGATCTGATGAAGAATTTGCCAATCTTCTCCGGTTGCCCACAA

TTGATGTTATCTCTGTGAGGCTGTGGTTTGATAAAAAGATCAGAATACCAAATGTTGCCAACGTTTGTTCTGGCTTTGAT

GATTCATCTGGCTTGACGTTCTTTGACCTCACCTCAGTATATGATGATTATTATGAGGAACCAATTACAGTCGTGGAAGC

TGAATTTTATGATGCTAGCCATTTGTTACCTCTAAGTGATGAACATATCGTATCTGAAGCTTCATCACGTCTTATAAAAT

GCATACAAGATTTTGATGGTGCTACTGTAATCCAGCAATCTGTCAGAAGATCTCCTAGTTCTGTCATCCATTATCTTCCA

GGTTTGTACAGGTATATGATGCGAGGATCATCTTCCTTTCCAAATTTGTTTCTTGCAGGCGATTGGATAGTCAACCGGCA

TGGGTCCTTTTCAAAGGAAAAAGCATATGTGACTGGACTTGAAGCTGCGAATAGGGTGGTGGACTATTTTGGTAATGGGG

ACTTTGCCAAAATAATTGCAGTTGAAGGAGACGAGTCTCATATAGAGACATTGCGGAGTCTCAATAGAAGAGCCAATGAA

CTGAAGTCTCAACTCCCTTTGTCAGAGTTGTTTCTCCAGTGATGTGATGAGAGTTCTTTTCTGTTATGACAATTGACTAG

TGCATATTATCAAAGGGTCATATCTATAAGGTCAATCTCTTTTCATTTGCTCTCTATCTTTTCTTCTGATCTTTGATCTG

AGAGGATGTGCCTTGTTGCTTCTATGAAAAGACTGTTTGCATATTCTTAACTAAAACTCAATACAGATCTTTCCAAAAGT

AATAGACAAATTAATTAAGAAAATGACTTTGGGATGAGGGCAACAGCATCATCCAAAATAGCAAAGATTTGTTTTCATTT

ACATGTAAATAGGGATCACCTAACTATCTGCTCAATAACACAACTTATACAGTGGTATGTATTAGCTTGTGCGTCTCCTG

TTAATAAAGGTAGCTACCTTTTCCATTGGCTTTGAGGTGCCTGTGGTGTATGCAGTACGCACCCAACAGAAATTATATTG

TCATACCCGTTGTGTTTCGTTGCTTTTCTTGCAATGGCATTCCCAGCATATATGGTCCACAGATTCACATTATATAGCAT

TGAAAATTCTTTTTGGTTCAAGCATATTAATGTTAAAGAAAATTCCAGCAAAATGCACTTTGTTAGTGAAAGTATCCTGG

ACAGGATTAGCTCCAGTTCTCAGGTAGGTTATATGTTTTCTGTTTATACACGCATTGCCCCGTGTTTAAGCCTGAACATT

>Cluster-342212.67768 PDS

CTTGTAAACGCTGAGGATTTGCAAGGAGATGAAGTTTACATATTCGTGCCTACATGTTCGAATTATTGATCTCAGCTACA

AAGTGGACCTGATGGCCACAAGCTCTAGCTCTTAGCATCATATCTTCTTTGATCATGTTCAGTACCTCAATGGGCTGCTT

CGCTATGTACACCTCTCTGCTCTAGGATGGGTTCGCCATTGCAGCTCTCGCGTCCCACTTGCACTGCCTTTCATAGCCTT

ATTATCAACTCATGTGCCAGGATTATGATATCGGAGTACTACATGGGACTTGCCATCTGCGGCAGGATACTGGATACCGC

GCGCGGGAGCCTTTCCATGGGCACGCCGTTTTGTCCCGTTGCAATTGGAGCATCCCCGTGCCGCTTGCTAGTGGCGTCGA

AAGTCGAAACGCCTCCTCACCTGCCCATTTTACGCATCAAAATCCTCTCGAGTTCCCTGCCGGAGTGGTATGATAGACAC

CCCTGGACAGGAACCTCCCCTCTGCCAAGAGACAGCCACGTGGCCTCCCAGCTCCACATTACAAAAAGGCTCGCCACGGC

AGGAGGAGGAAGAAGAGAAGAAGGAGAAGCACTGCTACACACGGCGCACGCCTGCGAGCTCCACACCGCGCGTCCTGAGA

CCGGCAAGCTAAGCCACCCTCGTGGGGGCATGCGCGGCCAGAGCAGCCGCCACTAGACGCTAGGATAGCTTGGCTCGATC

GCCGCGTCGGAACTCGCGATTTTGGACCTGCCTTTGGGCTGCCCGATTGGAACGGTTGGCCGGTCACCTGCGCTGCCACG

GGGACAGCCGGACAGGAGAGAGAAAAGGCTCTTTGTGCTCTCTGCTTACCTTCACTCTTCAGGTTAAACCGGACAAACTG

ACAGAGTACTAGGTCCGTTTCTCTCCGGCTTACTACCGATCACTGTGTCCTCTGCTCTCCCGCGGTCCCGCCCCTGCTCT

GTCCATCACTCGCTCTATATAGCCTTTAGTTTCAGCTAGTGTAATCAACTGGTCGACGCCACCTGCAGTTACGTTGCGTC

CCATAAGCCGGCCATCGTCATGCTTTCCTCCAGCCCCGCGATGAACTCGCCAGCGTGCGCTCGGCACGGCCTGTGTTCCT

CCGACGCGCCGAGCCGCAGGCTGACGACGTTCTTGGCCAGGCGCTCGACGAGGCGAGGCAGCAGCCCGCTGCTTCGGTGC

TCCGCTCGCGCCTCGGGCTCCAACACCATTGGCTGCCTCGAGGCGGAGCCATCGTGGGGCGGCGACGGCGGCGCGCCGGT

GCCAGCCCTCGCGCTGCCGGGAGGGCTCAAGGTGGCGGCGCCGGCGCCGGGGGAAGACGTGGCGTCCCCCGTGCCGTCCG

AGCAGAGGGTGCACGAGGTCGTGCTGAAGCAGGCGGCGCTCGCCGCCGCGCAGCCGAGGAGGACAACGGCAGAGCCTCAC

GAGCCGGTGGCGGGCGGGCTGGAGGCGGCGTTCGATCGATGCGGGGAGGTGTGCAAGGAGTACGCCAAGACATTCTACCT

CGGTAAGCGAGAAAGATACTGAAACATTTTCTACTAGGCTTCGATCTTGTCGATTTTGAATACTCTTATCAACTAGCTTT

ACAGTTTTGACAGTTAATGTTTTAGCTTTGTTCGAGGTACAAATCAGTAGCGAGCTTTTCCTTCTTCTTCTTCTTTTTTT

AAAAAAATCACAGCAGTATGACTGCCCTGCTCAAAAGGCCAAAGGAAAAAATAGATGATAAATTCCTGTAATATTCTTGC

AATAAGCGTCACCCACTGTCCAGTGAAGAACTCCCATTTCCATGATGATAAGTATGAACTCTACTATCAAGGACGCATTA

CCATGTACTAAATAGTCGTGCAACAGAGAACACGGTGTCTTTGTCAGAATCTTTTTATGTCACTGCATAGTTACCAGTCA

GCTTCATCGATGATCCTTAGCAATTCCGTGCACATGCGTGTCACTGATCTTATTTGTTGGTTGTATTGCAGCGACGCAGC

TGATGACTCCGGAGAGGAGAAGGGCGATCTGGGCAATATACGGTGAGCAAACCTAGGCCCCCACATGCCCATGTTATTTT

TCATGTCGTCGTAAAGGTTCCTTTCGTTCTTCACATGGTACACGACGAGCGTTGTCTGTCAGAC

>Cluster-342212.146951 LHCB2

GGGCATTACAGTCTACCCACATAAAAGATAATTCGTCTTTGAATTCAGTCGCCTCTATGACAATGCAACCAAAGGTAAAC

GAACACATAAAACTCATCGGTCTCAAAAAGATCGGGGTATTTCTTACGCATCTGCTCTTCAAGCTCCCAAGTAGCTTCAC

ACTCAATTTGATTACTCCATAAGATCTTTACATACTTGATAGTACAATTGTGCATTACATGCTCTGAGAACTCCAATATA

CGCACAGGCCGAGAGTCAATAGTTAGGTCTTATTAAAAAATTACTCGTTCAAACTCTATCTTCTTCTCTTGATCCTTTAA

ATATTTTCTCAACATAGAAACATGAAACACCGGGTGTACTCCAGCCATAGACTCATGCAACCATGCCTATGTCACATATT

TTTTTTCCATAAATGATGGGTTAAAGCAGCACGTTAATTGGCCATTCGCCCAGCTTGCGAGGCTTGCAAATTCCGAAATG

ACGAAAAATCATGGCTTGCGACTCCGATAAAGATCAATCATGCCTCATCACCGATCTTGCTCAGCCGCTATGCCCAACGA

CGAGACTGGGGGCCCCTATCGGTGATATGGGACAGGGGTCCCTAACACCATCCAACATTGCACAACAAGACATACCCGAT

CTCTAATAAGATAGGCCACAAGATTTAATAGAGAGAAATCTTTCCTCTACCTCCCTCTCTCCCACACATTACACAAGGTG

TGCATGCAAAGATGGGCCCACGTCCTTCTCTCTAGCTCTTGAGTTGTGTGTCGGAAGTAAAGGGCTCAAAGCCCAAAGTA

GAGACAACAACAATAGCATGTCACGAAAGGCAAGAGGGGGCATGAGTCCTTCTCTACTGCACACTCATTTACTCCACTCT

TTATTGTTTAACTGTGAGCAGCCTCTTCATTAATAAAAAGGAGCGCCCGGGATAGGGGAGGAGACTTCTTCCTCCTCCAG

GACTTTAATGAGGGCTCCTCTCAAGCTCTCCTCATGTATTCTCCACCCAAAAATACACAGCAATACGGAGAAGCAGAATG

TAGGGCTATTAGCCTTCGAGCGGTCAAAATCTAGGTAAAAAATCCCTCGTATCTTATTTCTTTAATTCACTAATCTTTTT

AATCATTTTAGAGATTATAACGTATTCATCACCTAACCGAACATCAAGGGGTGATCTTGCTAACCTTAATATCCACCTCC

TATTGACAGCGGACATAAGCCATGTTTTCATCGTCTTCAACCTCCAGCGTCTTAGAGTCTCTATTCCATGGCCGGCAAAA

TCCGGGGCCACCAGATGCGTGGCCGTGCGTAAACAAGATTTTCCGGACCAAAACTCCTCCAGTACGTTCCTGGATCCTGA

GATTACAATATTTGCATTCATATTCATAGTACAAATGTAATGTCACAAACGAGTAGTCTACAATCGCGAAACGGGCCCGA

TAGCTTAATTAAGACCCTGGCGCGAACTTGGTGGCGTAGACCCACGCGTTGTTGGCGACAGGGTCGTCGAGGTGGTCGAG

CAGGTTCTCGAGCGGGCCCTTGCCGGTGACGATGGCCTGGACGAAGAATCCGAACATGGAGAACATGGCGAGGCGGCCGT

TCTTGATCTCCTTGACCTTGAGCTCAGCGAAGGCCTCTGGGTCGTCGGCCAGGCCGAGGGGGTCGAAGCTGCCGCCTGGG

TAGAGCGGGTCGACGACCTCACCGAGCGGGCCACCGGCAACGCGGTACCCCTCGACGGCGCCCATGAGCAGCACCTGGCA

GGCCCAGATGGCCAGGATGCTCTGCGCGTGCACCAGGTTCGGGTTGCCCAGGTAATCCAGCCCTCCCTCGCTGAAGATCT

GTGCGCCGGCCTTGAACCACACGGCCTCGCCGAACTTGACGCCGTTGCGGGCGAGCAGCTCGGGGAAGACGCAGCCGAGC

GCGCCGAGCATGGCCCAGCGGCAGTGGATGACCTCAAGCTCGCGGTTACGGGCGAAGGTCTCCGGGTCGGCGGAGAGCCC

AGCGGTGTCCCACCCGTAGTCACCGGGGAACTCGCCGGTCAGGTATGACGGGGTCTGCTCGGAGAACGGGCCAAGGTACT

TGGGACGGTCAGGGCCGTACCAGATGCTCTGGGGCGCGCTCTTGACGGTGCGGCGCATGGTGATGCGGCCGCCGGCATCG

ACGCCGACGCGGCTCACGAGAGCCTGGCCGAGGAAGCTGGTGGTCTGCTGCAGCGCGGAGGTAGCCATTTTCTTGTGTGA

>Cluster-342212.97612 LHCA2

CCTCCTCCTCCTCCACCACCGCCGTCGCGGCCCTCCCCAGGAGCGGCGTGCGCGCGCCGTCCTTCCTCGGCGGCAGCAGG

CTGCCCGCGGCGAGGCCGTCGTTCGCCGTGCGCGCCGCGGCTGACCCCAACAGGCCCATCTGGTTCCCCGGCAGCACCCC

TCCGCCGTGGCTCGATGGCAGCCTTCCCGGCGACTTCGGCTTTGACCCATGGGGTCTCGGATCGGACCCGGAGAGCTTGC

GGTGGAACGTGCAGGCGGAGCTGGTGCACTGCCGGTGGGCCATGCTCGGCGCGGCCGGCATCTTCATCCCGGAGTTCCTG

ACCAAGATCGGCATCCTCAACACGCCCTTCTGGTACACCGCCGGCGAGCAGCAGTACTTCACCGACACCACCACCCTCTT

CATCATCGAGCTCATCCTCATCGGCTGGGCTGAGGGCCGCCGCTGGGCCGACATCATCAAGCCCGGAAGCGTCAACACCG

ACCCCATCTTCCCCAACAACAAGCTCACCGGCACAGACGTCGGGTACCCTGGTGGCCTGTGGTTTGACCCGCTCGGCTGG

GGCAGCGGCTCACCAGAGAAGATCAAGGAGCTGCGCACCAAGGAGATCAAGAACGGCCGTCTCGCCATGCTCGCCGTCAT

GGGCGCCTGGTTCCAGGCTGAGTACACCGGCACCGGCCCGATCGACAACCTCTTCGCCCACCTCGCCGACCCCGGTCACG

CCACCATCTTCCAGGCCTTCACCCCCAAATAAGGAGTTGGAGTTGGTGATGATTTGAGGAGGAATGCTACCAAGCATGCA

TGGGGAAGAAGGGATTGTCATATTTTTGGAGGGATTTGACTGTGAATGTATGGTTCTGTGGAGCAATGCATGCATGCATG

TTGGTCGTGTACTTGTACTTTGGAGTGACTGATGTAAATGTAATACTCATTAGAAGTAATGTTTTATCTTCACATAATCG

CAGCTATTGATGATATCCTACATGAACAGGAAACACGTTATAGCCGATGAATAATTTAACCATGTGCCAAATAACCCAGA

TTAATTGGTCCCTAATTATAGCTGCTTCCAACCCAAGGTGTCGTTTCATCATAAAAGGCCAAAGAAACATATGGGTTCTC

CAAACAATGTTGAATGTATAGTGCAATTCTTGTATCAAACATGGAAATTTCATGATGCAATTTTTTGCCAACAATGGGTT

CAGATTTAATAATAAAATTAAATACTAATTTTGTGTCAAACGATGTTAAACTTGTTGTATTTTTTCCTTAGAATAACATT

TCCTTTTTCTAATAAAAACGTATTTCTGCATGGAGAGTCTTAACCCGAACGTAATTTTTGGTGTTCCGTGGGCCACGCTA

TTTGAATGGGCATAGGGCATACAAAGCTGGTGGTTTAGATTGATT

>Cluster-342212.33835 PGR5L1A

GACTACTTGAAGATGTTCCTGCTCAATGTTCCAGCGACTATTGTTGCTCTAGGACTGTTCTTTTTCATCGATGAGCTGAC

TGGCTTTGAGGTCAATGTATTCCAGTTTCCGGAGCCCTTTGGCTTCATATTCACGTATTTTGCCGCTTTGCCTCTGATAT

TGGTTACGGCGCAAGTGGTAACCAAGGCCATAATAAATGATGTCTTAATCCTGAAGGGGCCGTGTCCGAATTGCGGCACT

GAAAATCTTTCCTTCTACGGGACGATACTGTCGATCGAGAGTGGTGGAGCAACAAACAATGTGAAGTGCGCAAATTGCAA

AACAGTGATGGTATACGACTCAAAAACTCGGCTGATCACACTTCCAGATTCATGAAGAGGATAATTATAGTGGCACTTCA

TTAACAGGGAGGAGCAACGAGATGCAGAGTTTGCCATTCATGCTTGGTAATTAAGTGAGCAAATGGAATTGTCAAGTCTG

CAACAAACTGCTAGCTGCTTCTGTGTTGTAGTATGATTTTCTGTAGATATGTATTTGCAATTGTACCTGATTCA

>Cluster-342212.33834 PGR5L1B

GACTACTTGAAGATGTTCCTGCTCAATGTTCCAGCGACTATTGTTGCTCTAGGACTGTTCTTTTTCATCGATGAGCTGAC

TGGCTTTGAGGTCAATGTATTCCAGTTTCCGGAGCCCTTCGGATTCATATTCACATATTTTGCCGCTTTGCCTCTGATAT

TGGTTACGGCGCAAGTGGTAACCAAGGCCATAATAAATGATGTCTTAATCCTGAAGGGGCCGTGTCCGAATTGCGGCACT

GAAAATCTTTCCTTCTACGGGACGATACTGTCGATCGAGAGCGGTGGAGCAACGAACAATGTGAAATGCGCAAATTGCAA

AACGGTGATGGTATACGACTCAAAAACTCGGCTGATCACACTTCCAGATTCATGAACAGGATAATTGAAGGTAGGAGCAA

CGAGATGCAGAGTTGGCCATTCATGTTTGGTAATTAAGGGAGCAAATGGAATTGTGAAGTCTGCAACGAACTGCTTGCTG

CTTCTATGTTCTAGTATGATTTTCTGTAGATATGTATTTGCAATTGTACCTGATTCAGACGGATTAAATTGCAGAATGTA

TAGAAAATCTAGATGGTTGTGAGCCTATGACATTAAACCGAAGCTTATACGTAGTCTTGGCCAATTGTCATGCGCCACGA

ATAAAAGGGGGAAAAGAGAGAGATGTGCTTGCAAGGGCCATTACATTTGGCAACTGGACCATGGGACCATGGCTTGTTTT

GGTTTTTGTCTAACGAGCTTGCGTCAAATCACATCGT

>Cluster-342212.200986 PGR5

TTCAGCTCCTCTGACAACATGCTATCGTCGTCATCCTCCTCGATGCTTATGAATTCATCACTGGACTTGCCGCCGTCACA

AGCTCCTCTCTCATGGTTCTCGTCAGGTGCGTCCATGTACATGTGGCCTTCTTGATAATTCTCAGCAATATCCTCTCCTT

CTTCTTGAGCACCCACTGCATTCTGGACGCTTGCCTCCTGATGCGGACTGAACCTGTTCGTCTTCATTAGCACTCCAACT

GCATGTTTTGCAACGTCGATAGCCCAGTCCCGGCCCTCGCAGGCGCCACTGTTCTTGGTATCATGTTCCGAGACTTTTTC

CTCTGCTTTTGTAAGTAAGTCTAGTGCACAGGTCCTGATGTTCTCGGAGGAGAAGCGGCCGAGTTGTGCAAATTCTAAGA

GAAAATGAACATCTAGCTGGATCTGTTGAAAAAGTGTGTCCTTGGGAATGCCCTGGGCGTGATGGATCCAGGAATCTAAG

TTATTAGAGAGCCAGAAAATCATTGCCTCCATCAACTCCTGCAGCAGCTTTTTCATTGCTCCATCCTTGCCAGTTAAGAT

GGTGCCATACAGATTATTCAACTGCCTCACCCGTCCAAACAGAACCTGGAAAGGAAAGGAAGGCATCATGCCCTGTGGTG

AGCTAGCTATTCCTTCTCCAGATGGCTGTATGCTTGACATGGTGTCTCGTATAAACTGCTGGCAGAAACAGGTCCATACT

TGCCCGGCTGCTTCTTTAATCAACACGATCAAACTGTCCAGATATCCTTTTGTCAGGAAGTCGGTAACAAGTGAAGGTTG

GTCGCTCTTGAACATGGCATGAGCAATGATCGGAAACAAGGACACTAAAGTTGTGCAGTTGATTAAGATGGAGAGTTGCC

ACATGTACTGATGATCTTCGTTTTTGAGGGCGACTTCCTTCTCGGGGATGAGCTCTACAATGGAGTGCATATACTCCCTG

AACAGCTCTGCAAGAAGTTGCAGGACCGAATTGTTGATTCCAAGTTCTAACAGCGGGTACAAGTCCTCCACAACATCCTG

TATGAGTGTAAGAAATTTCCTTCCACTGGCTGTGAGCAGGCAGTACTCGATGTGCAGTCCAGTGTCCACAGCAGGCGATA

CCAGCAGTATTCCAGAGACGGGGAACCTACCGAGCACCCAGGAGTCAGACTCGACAAACAATCGAGTGACCTCCTTGAGA

TGCCTGGCATACACGGTCAGGACCTCATGGAGACAAGGTGCGAGGAGATCCAACAGGTCTTGCTCGGAGGCGATGACGTG

GAGAGGCCTCAGCAGGGCACAGTAGGAGTGTGCGCACTTTGCAGCTTCCAGTGCCAGAACCACACTCTGGCCAACCTGCG

ATGTGGATTTCACGTACTCGGAGAATGCGACGCTGAAGTCCTCCATCTCTTCCCTGGCCCATCGGATGAGCTGAGGGGTG

TAGGGAGATGGATGCCCGTGCAGGGATACGAAGCTTCTCGATGCTTCTAGAATGGAGGAGAATACCATGCACGCGAGGTC

CTTGATGTAATTGTTCTTCTTCTTCTTCTTCTTCAGCTGGTGCTGTTATACGAGGCGCAGCACAACAAGTAGTACTAGTA

GTTCCAGCAGAGACGGGCGCCACGCGCTGCACGAGAAGCGGCATGGCGAATTGGCAAAAACTGGTTGGTTTAATTAAGCT

GGTACACAGCAGTAGTGCAAGACCAATAATTCGATCGGACGGGAAGATA

>Cluster-203172.0 TPT

CATTTCTCGAAAAATTTTAAATTATAATATCGTAGTTATTAGTTATTAAATTTGATGCCGTGTGGCCGTCTTTATCGACT

TACTCGCTCGATCGATGTTTCTCGCTTGATTGTAGAAAAGGACACCGAATATTACCATGAATGTCCCAATTGCGCTCATG

TAGCCAACTTGATTTTGGAAAATCAACACGGAAAACCAAATTAATAAAGCTCTTTTAAGAGTGTTACAAACACTATAAGT

CACTGGTGAGATATAACTCATAAGAGTAAAGGCAAGCAACGTCTGCATGTGAAACGAGACGCCGTTAATGATGAAGAGGA

ATAAATTTACAAAGCCGAAGCCCTCCAAGCTCAGATCGACGGTGAGTAAGCACATGGGCACTAGAATGACAACTGACGTA

ACGCTCGAGAAGAACTGAACCTCGAGAGGCGTGAGTTTAAAATGATCACCGCATAATAAAAGTTTTGTATAAACAATTTG

TACACATTCGGTGAAATTGGTCGATATGGCAGCGATAAAACCAAAAAGACTAAAATTGATCTCGAAAGAACTGCATAAAG

CTAAGCCGAACATGATCGGAAATAACGAAACATTCGTCCAAAATCCGGTGATTTCACCAATAATGAGACGAGAAACAATC

ACTGTAAAAATAGGCGATGAGCTTTTGACAGTCTCAACAAATGAGATCGCCGCTTGTTTGAGTGCCATAAGACCAAACAC

AATTGAAAATAACCTAAGAAGACCAATTATAAACATATTCTTGAGAAAATGCGGTGAGATGTAATGCTTGTTTGGACTGC

TAAGAGACGGAAGCTGATAGGATGGATGCGACTTGTTCCAAAGAGTTTGTCTCAGCTGAATGTAACCGGAGATACTTGTC

ACAATCATTTGAATTGTGCCGACAACAAACGAGTCACTTTTCCGGTTGCTCACAATGAACTTATTCGTATACAACGTGAG

CGCGCTGAAAACATACCAGACAAATAGAAGAAAGAGGACGAAATGATTGGCGAGGCCACCTTGCCTCTCGATGATCACCG

TTTGAGATTCGACCGCGCTCTCGGCTTTAGTCGCATTGTGGAAGTCCTCATTCAGCGGCTTGTCTTCGATCGCATGCCGA

AACTCTCGCAGCTCGCCTTTCAGGAACGACTTGAGATCGACGGACATTTTGGGCGACGACGGCACTCTATTAGTACTCAT

ACCACCACTACTACTACTATTACTATTCACCTCTCCGTTAATTATCACGCCATTT

>Cluster-342212.172526 SPS

ATCAACAAGAATGCCATTATCAAGAACCCGATGTATGTCAACAGGCCCACCATTTCTGGTAGCAACCATTGGTAGACCAT

ATGCAGCAGCCTGGCAAGTGACAATGTGTATACATCAAACAACCATCATTAAGGCAAGCATTAGAGACCAATGTAAATTT

TGTAGCAGCAGCAGCAAACCTCAATCAAGGTGAGCCCAAATGGTTCAATGAATGCACAATTTATAAAAACCCCCTTTGTT

CTTGCCGCTAATCGATAAATATCAGGAACTTCAGATTGCTTATGGTGCTTGGGGTATGCCACTTGTCCATATAGATCATA

TTTGTCAATTAGCTTCAGTACTGAAGTCAAAACAGCTGCATTTGTGCTTGACATTTCATCAATAACATCACGATTACCCA

TAATTAGTGTAAGGTTTGCTAAATTTCTCAGTTCCCGATGTTCACCAAATGCCTTGACCAATGTAGTGATATTTTTCTTG

GGATCTGGACGAGCAAGTGCGAGAATCATAGGCTTACGGGGGTTTGAGAAGAAACGCATAATATCAGCCCAAATAGGTGG

ATCAGGTGAACCTGAGCCATCTTCGCTTCCTTCTTCACCATCCAGGTCAACATCATGAGGGGCTATATGGCTAAACTCCA

TGCCAGGAGGGATTGCAATCATGCGAGGCATGTAACGACCATAGCAGCTCACACCACGTTTTGTTCTTACTCTAAGTTTT

CGAGCCATAGTCAGATCAAAACCGTCATATAAGCCCCATTGTTGTTCAATTTCTTGCCTCGTGCTAGTAATAATGATTTC

AGATGCATCAAGACAAAGTTCCTCAGCCTCAATTCGACGCATTATCTTGTAAGTGGTATTTATTTCATCTCTCGTCTGAC

GCCCTTGCTTCAGAAGCTGCTCCAACTTGTCTCTGCCTAGAGAATGACCTGTGAATACCATGGGCACATTGAGTGCCCCA

GACAGTAAAGCAGAAGAATCACCAGCGTCAGCATAGTGTCCATGTATAACAACAGGCCATACTGGCTGCCCACTACCAAC

TTGTTCACCAAGCACCTTGGACATCTGCATGATATGAACAAGCGCACCATCAACGAATTCCTGGATGTGGGGCCAAAGAT

GCTCCTTGGGGATATACTTGTCCCTTGGTCCAAATGGTATTCGGACAATATAGGCACCACTGCTTTCACCCATCTCATGG

CCTGCATCAGAACCTCTTGGGCTCAGCATCTCAGTAGGTTCCCCGTAGCTCCAGTCAACATCTGGTGCAGAAATCTGCCT

TGTCAGTAAATCCACTCTATATACACCTGATGTTGAACCCAAAGCCCTAGCAAGCTCAACAACATATTTCACCTGACCAC

CCGTGTCTGAATCACGACCAAGCTCCATATTCTCACCGCGTACCAGACCGTGTAGACTGATCAGTACTATGTACAGCTTG

TCAATTGAACTTGTCTTAGGTGAGCTCCCAGTGGTGCTGTCACCATAGGCAACAGATGGATCGCCAGCATCTTCACCCTT

TTCTCCTTCAAACAGATCTTCGGACATATCTGCATTAGCATCAGTTCGTGTTTTCTCTGTTTCTGGAAGGCGCTTCAACA

AACGACAGGCTTCTTCTTTCTCAAACTCCTTCTTCTTCCT

>Cluster-342212.147485 SUT1

GGTGGGGCCACAGCTATGCGCGCCACGGCCTCTTTAAATGCCTGGCCATCTCGCTCGCTTCGCTCCCAACACAACACCTC

ACACACCTCCTCCTCCTCCGCGTCTCCAGCTCCTCCTCACTCCACGCTCACTCGCGCAACACACACCTCCCCTTTCCAAT

CTCGTCTCCTCCGAGACTCCAGACGTCTTCCACCAGGAGAAACCCAGCAGCTCCTCCACCGATTTGCCTGAACGGCGGCG

GCCTGATCGTCCTCGGCCGGTGATCGGCGGCGGCGTATACGGTCCTGACAAGAACCGTGCGCGTGCCATCATCATGGCGC

CTCGCGGCGACGGCGAGCTCGAGCTCTCGGTGGGCGTCCGCGGCGGCGGCGGCGGTGGCGCCACCAGTGAGCTGGCGGAC

CCCCCGGCGTCGATCAGCCTCGGCCGGCTCATCCTCGCCGGCATGGTCGCCGGCGGCGTGCAGTACGGGTGGGCGCTGCA

GCTGTCCCTCCTCACGCCGTACGTGCAGACTCTGGGGCTTTCGCATGCCCTCACTTCATTCATGTGGCTATGTGGTCCTA

TTGCTGGCTTAGTGGTTCAACCATGTGTTGGTCTGTACAGTGATAGGTGCACTTCGAAATGGGGAAGAAGGAGGCCATTT

ATTCTGATAGGATGCTGTCTGATTTGTGTTGCCGTTATAATTGTCGGATTTTCGTCAGACATTGGAGTTGCTCTGGGAGA

CACAAAGGAAGATTGCAATCTCTACCATGGTCCTCGTTGGCATGCAGCAATTGTATACGTGCTGGGATTCTGGCTTCTTG

ACTTCTCCAACAACACAGTGCAAGGTCCAGCACGTGCTCTAATGGCTGATTTGTCAGGTCGGCATGGCCCTAGTGCAGCC

AATTCAATCTTCTGTTCCTGGATGGCATTGGGAAACATCCTAGGATATTCTTCTGGTTCCACAAACAATTGGCACAAGTA

AGTGGATCTGCGTGTACATAAATTCTATTTGGCTCTGTACCCTGCAAATTTGTTTACTGATAATGAGGTGAATTCTGCAT

AGGTGGTTCCCCTTCCTTCAAACCAAAGCCTGTTGTGAAGCTTGTGCAAACTTGAAAGGTGCTTTTCTGGTGGCAGTGGT

AAGATTTACTGTACCTCACGATATTCCACCTATGTTACAGTGCATCATTTGTAACTCAAAATGCTAAAACTTGGCCCTTG

TGTGTTTTCAGCTTTTCCTTACCTTGGCTTTGGTCGTAACCCTGATCTTCGCCAAGGAGGTACCATACAAGGGAAACCAG

AACCTCCCAACAAAGGCGAACGGTCAGGTTGAAAGTGAGCCCACCGGGCCACTTGCCGTG

>Cluster-342212.45411 SS

GATCTGTAGGGGACTACACAAAGCTCATTTCACGCTTCTACAACCAATTAGGCCTCTTCAGCGAATACTTAACGCAGAGC

TGATCCAAATGATATCGAAGAGTTTGAAAAACATCAGGCAATTGCCTCAATTCATGATCCTGCCCTTGCTCGAGCAACTG

CTCTTTGATAGATCTCTTCATACTGTGAAGCCGAAGAATCCCAGCTGAAATCTATCCGCATGTCCTTTTGTACTAATTGC

TTCCAGACTTCAGGTTTTCTATTGTAGTAGTTGACAGCCCGTTCCAGTGCTCCGTTAAAGCTCTGCTCATCAGCATTCGC

AAATGTAAAGCCATTTCGCAGCTCCATGGGTATAGTTTCATCGTCAAAGTCAAAAACACTGTCATTCAAACCACCTGTTT

TACGAACAATTGGCACAGAACCATACCGCATGGCTATCATCTGAGTGAGGCCACATGGCTCAAACATAGAAGGAACAATG

AACATATCTGATGCTGCAAAAATCATATGTGATAAAGCATCATCATACTTCAGAATCAACCGGATATTGTTATTGTTCTG

AAAATGGTCTGCAATACCCTCAAATTCCCTTTGAATATGAGGTACTGGACTTGAACCCAGAAGAACAAATTGCCCGCCCA

GTTCAGCTGTTTTGTAGATAGCATGTCTGATGAGATGTACACCCTTTTGAGGAACTAGCCTTGTAATGCAACCAATTAGC

GGTTGGGAGGCATAAGTGGTAGACAGTTTTAGCTGTTTTCTGATAGCTGCTTTGTTTGCTGCTTTTCCATGTAGATCATT

TGCGCTATATTGAACCTTGATAAACCTATCTGTAGAAGGATTCCATGTGTCTGTGTCAATTCCATTAAGTATTCCGACAA

ATTTCTTGGAGTGTATTTTAAGTGTGTCTTGGAGTCCACGCCCACCCTCTGAGCGCACCTCTTGAGCATATGTTGGTGAT

ACAGTTGTGACAATGTTGGAGTAAACAATTGCACCCTTAACAACATTTATTCTACCATGTGAATTATCTTGCATTCTGTC

TGGTCTATCGAGGCGCTCAACATCAAGGCCACAGAATGCTAAATCCTGAGGCGGAGCAGTTCCTTGATATTCAAAATTAT

GGCAGGTGAAGCAAATCCTAGCCAAGTTGAATCCCAGATTTGCATATATATCCCAATAAAGGGGTGCCTATAGAAAGAAG

AATAACATGATATGCTCAAGAAATATACTGAAAACTAAGGAAAAGAAGAATGATTTTCAGTCCACCAAAGGTTGTCGAAT

CTTACAACAAATCCAGTCTGCCAGTCATGGCAATGGATTATGTCAATTTTCTTCCCAGACTTGTAGAGTAACTCCAGTGC

>Cluster-342212.241093 GWD

GGCTGCATCATTTTCATAAAGTAAGGACAAAATCACAAACCCTAAACAGTGGGGGTAAAATAACAATTAGAATTGAGACT

TGTGATAAGTTAGAACACACTTGCCCCTAAAATTTATATGGGTTATGCACCCTGCGTCTTCCTATTTATTCCCTTTCTAA

ACAAGGAAGACATAAATTACATTTGTGGTCTAGTCTGGACAACATATATCTTTCCATCCTTCACCACTCCTTCAATGTCC

TGCGGGGACCCATACAGCTCCTCAATCGCGTAGCCAGTACGAGCAATGCTTGAGAGGATTGAGTTGCGGAAGCTACTATC

TATGATGAGTGGATCAGTTGTGTAGTCAAGAACAACTTCTTCTTCCTTATCCATGGGCACACTATCATAGAGTCCTGCCC

CAGCATAGCCTTCCAAATCCTCACCATTTGAGTCAGAACGAAAGATGATGGATTGCTTGATGAAGAGACCAATTGGCTTG

CTTGGATAACCCAACACCTTGGGAGAGTTTAGGTCATCCTTCTTGCAAACAAAACTCAAGGCACGACCAGGATAGGCTCC

CACAAGGGTCTCCCCAAGTCCTTTAACAACTTCAGCGTATATCTCCAGATCATCTCCAGATGATGGGTTGGTGGTATGGA

TCACAAAGGCATAGTCTGCACTAACAATTTCTTGCACAAGAACTGCCATTGAGAGGTAGTCATGATCAAGCTTCACTTTA

CGAGTGCTGAAGTATGCTCTTTCGTTCCATTTTGAGGCCCAGACCTTTTTAATTGCCTTCCACGCTTGCTCCCAACGTTG

TTCACCTTCATCTCCTGGCCAGGGCATCCCAGAACCTTGCATCTGCTCCTTGAGCTCTTTGACCAGACTGACAGGAGCTA

TCAGGTTTAGAATAGCATGCCTCATTTCATTAAGAACACTAAATTCTCCTTGGTTGAGTTTTTTCTTCAGACTCTGTACA

TTTTGGTCTACTTCCTTGTTTATCTTATCAGACAAAACTGTTTCAAAGGTTCCAAATGGGAGTGCTACTGATGTTGGGAC

TCCAACGGATGGAGGGACTTTCCCTTTTAGGTATGCTATATTTCGTGATTTAGCTCCAACCATCTCATCAGAAAATTCAT

CTGCAGATATTGCATATTTTCCTGGAAATTGCTTCTTGACTAGTGAAATAGATGGTCCTGATTGATCATCTTGTGAAGTG

GGGGAACTTGTAACCAGAAGTTCACTCTCTGTAGTTTCTCTGTAAGTTATATCAGCAGAAGTAGGTTTCAATGAAAGCAA

CCTCCCCTCATTACGTTGAAGTTCAGATAAAGTTTCCGGGTCAAAGCATGTTGCGAACAAAACCTTCCTAGGTTTGCGAT

GTTCCTAAGAACAGGATCAAGACGATTTAGAAGTAGGGACAGTGAAGCAGCTGATCCACTACGAATGATTTCCTCTGTAA

ATATGCTAACTGTCCATTTCTCAACTCCGAGCAAGGAACCAAGGTATTCAACTGAAGGCTGAAGAGTCTGATTGTATTCT

TCTCCCTTGGTTACGAGTGCAAGTCTAGTTCTGTCAAGACACGCTTTTGCATATAATGCCCATTGGTTATCCGAATTCTT

AGCCATGCTAAGGGCCTGGTTCCACCCCTTTAAGCAGCATAAGAGGTTCTCATTGTCTTCAGTGGACAAAGCAAGATTTT

CAAGAACCAGGCTGATGAAGTACATGATTTTCTCAGGTGCTGCGTTATTCAGTTGCTCATATGACCTTTCAACTGCTGTC

CTAACAGTAGAATCAAGAGCGATGTCCAAAAAGATGAGATCCTTCAAGCGTGCTGATGAGCCACAGAGTAAAGGACGAAT

TTCAGCTCGTGCCTCCAACAAAGCTTGAAATCCGGATGGCAGTTCCT

>Cluster-342212.177252 PWD

AGATACCTGAATCCGAACACACCCCAAGTCTCCAGTTTTTATTTATTTATTTAGATGGAGGGACAATTCCATCAAACAAA

GGCTGACGCCCAATCGGTGGCCACGAGATGATTTACGCATCAAGATTACAATGAGGCCAGATATGCCCTAAGCTTTACAT

GATCTCCAGGCTTTTAGGAGTGAGCCATTTTTGTTGGAACCAAATCCTTTGCATTGGGTACTCCCCAATACAGAGAGGCA

CTGTAACTGGCATTAGGCCAGCTTCACAGCCGTCAGATCTGAAATGAATGCCCCAGATTCATGGCATTAGAACACTGTAC

CCTCACGAATCACTGTAGCTCCCGGCATTGAGTGAAGTAATGTAGAGAATTTGGATCCATTTTTGTTAGATAATATATGA

CCGGTGCAGTTTGAGCTGAAAGCTGCTCTAAAAGCTCTGCCACAAGCTTAAACACGGTTACACTAGATTGAAGTACCTGT

ACCTTACTATGAGTGTTCCATACGCTAACCTCCCATTATACTTATTCCTCCAACATTTTTGCATTAAAAATTCATTATTT

ACATCCAAAAACTTCACTTGAGTAGCGAAACCAAATTCTATTACCTTTTCACACATTGATACAATCGAATGGCTGGGGCT

GGTATGACTTTTTATTTGACCCAAGGCAAGAAGATAAAGGGTCTTGAATCACTGCTGTGTACTAACTACAAATTCCACAC

ATTTCTCCAGCCATGCATTTATTTTATTCACCAAAACAAAAGGCAACACAGATCACAGCAAAGCTTTTACTACCCTATTA

TGGCTGCGGCCTGCTCTGCACTATGAAGATGTCTTTGCCGATCAGGCAACCTTCCACATCTTGGGCACTTCCGAACTGCT

GCTCCAGAAACTGTCCAACGGCAGCAAGCCGCTGACCAAACTGCTTCCTAAAGGTTGAATCAACCGACAATGGCTTCTTG

CTGTAATCTACAGTAAGACGAAACACTTCCCCATTAGCAGGACCAGAGTTCAGCACAACCATCTCCTCGCTGAAATTTGC

GAAGGCTAGAGTGCTGACTTTGCCATTAAACTTGTCACATGATAGCCTCCATGGGGTCCCGCGGGTTCCAGAAGCGAGGG

TTTCTCCCAGTCCAGGGGCGACCTCAGCTTCTACCAACTTGGGATCATGATCAGCTGGGCTTATAGTGTGAAGCACAAAC

GAGAGATCTGGCTGCAGCATTTCTTGCACAAGAATGGCCATCTTGGCGTCTCTTTGAGGTACACCAGCGGCTCGTCGGCT

AAGTATTGCTCTTCTTGTGTACAATGATGCCCAAACTTGTCCAACCGCAGCTCCAAAGGCACATGGGTCACAAAGGCTGA

CATTAGGAATCGATTCATAGAGCCCAGCAGCTGACATACCAGCCAAATCCTCGACATTAGCACTAGATCTGACAATCAGT

CGAGCATTCGGGTCGAAGATATTTTTTAAAGACTCAATTGTTTTTTCAGATGGAGAAAGAAGTGAAACTATAGATTGAAG

CTCTGAGGATAGGCTATCAAGTTCACCATTTTCAATTATAGCTGTTTCAATCCTTTCTAGAAGGCTTGTATACGAATCCA

GTGATCCACTATTCTTGAGTGCATCTTCCATGTATCCAAATGGAATTACAGCACCTGCTGGAACTCCAAACGCTGCTGGA

ATTCCTTGATCATTGTAAACTTTATTTGACAATGACGCCAGAACCGAAAGGGTTCCACATGCAGCAGCTTTTGCACCAGA

TGATTCGACCGATGCCTCTGAAAGCTCAAGAACACCAGACATTCCATTCACTCCCGAAGTGTAGCTCTTTAGCTCAGACA

CATCTAGCGACTTGTCTATAGCCAGTGGCAGAGATAATTCTTTTGGTAGCTCTTGAGCAAATAAGTTCCCTCCACTAGAT

GGCTCACTAGATGGCTCTGAAGATATGGCACTGTCATATTTGTCTGAAACTACTGACAGATCAACATTATTGGATGATGC

CCCCAACCTCACATGTTTTCCCTCAAGTGACTTTATGTCTGCAATTGTATCATCATCTTCACAAGTTACAAATATGACTT

TCTCTTGACGAGCTCTAACACCAAGATGCGATAGGTGAGGTAACTCTTGTAGAAGAATAACACCCACTATGTTATCTCCA

GCAGCTTTGACCTCTTCATCTCCATCAGCCTTATTTACAACCAGCACAACAGGCTCTTTGATGGATGGGGGCAACATTCC

AGGAGCTATTCGTTCAACCTGTATCAGAGCTCCATGGGCCACTCCAGGAACAAGAACATCCCAAACCGATGATCCAAGTA

CTGCTCGAGCAGCTTTCAAAAGTACAGTGCAAAGTTTGGAGACCTGAAAAATAACACCAGCACGAATTTCAGCTTCAGTG

TATGTTCTCACACTGTTCTCTGGTATTCCAAGGGCTTTCCCAAGAACCTCAACTTTTTCAGGAAATATAGAAAGTAGTGC

TTCAGAATATTCCTCTGTTAGTCTTCTTCCTCTATCGAGAGTAGCTTTAAGCCTTAATAGCCAGACACCCTTCTCATCCT

CACTGCCTTTATGCTTCCAAGAACGAAGTTCATTGCCTATAGCAGTACATTCCTCCGGTTTCCAACCTGAAAAGCTAACT

TGATCGATGCCAATGATAAGGGCATCAAGGGCATCTTCCCATATACTTTCATTATTAGCCCCACCCTGTGCAAGTGAAGC

TGATCCACCTAGAGCTTCGAGTGCATTGATGTACCTGCTTAACAGAACAAACGAATAGTCCTCGAGCCCAATTTCACAAA

GGCGCCACTTTTGTCGCATTGCTATAGCACTATCTGGCGCATCTTTCCTAAGTCCACTTTCAAGGCCCTTAATTAGAAAT

GATCTTAGAGAAGATAACGCTTGCAAGGTCTCCATCAAAACTTTAATGCTACCATTTTTATCCAAATGTTTTGCATCTCC

TGATTGGTCCAAACTCTTTTTGGTATTCACAAAGGATGAGAGAACCTGTAATGCAGACTCACTTAAAGATTCCTTGATGG

ATTCAACTTGCTCCAATAGGCTGCAATACCCAACTTACAGTATCAAATAACAACAGAAAACTTTATCTAACTAATGTGTA

CAAACATTTGATGATCATCACAGGAGTTACCTTTAAACCTTATTCAGTGAAATATCTTCTCTGTTTAAAGTGTACATTAG

>Cluster-342212.182369 BAM

ATTTTTTTTTTTAAAAAAAAAAACCTTCTTCCATCTTGATGTCATTTGTGTATGAAACTTGGGGCACGATGTTTTGAATG

TGAACATCGAACTGAACACTGATTCTAGCTTGCAAATCAACTGTGTGCAGCATACCACTTCCGAAATGGGTTTTGGAGGA

GATGGACAAGGACCAGGACCTGGCTTACACCGACCGGTCGGGTCGCCGGAACTACGAGTACGTTTCGCTCGGCTGCGACA

CCGCGCCCGTGCTCAAGGGCCGCACCCCCATCCAGTGCTACGGCGACTTCATGCGCGCATTCCGCGACCACTTCGCCACA

TACATGGGCAACACCATCGTCGTACGTTCTTCACTTTCCTGATGAGAATTACGCGTTTCCGAACCACATCATTGCTTGTT

TCTACGATTGTACTCACCATCCATCAATCACCGTGCAGGAGATCCAAGTTGGCATGGGTCCGGCCGGCGAGCTGCGCTAC

>Cluster-329512.0 FNRL2

GTGATTTCACCCAGCCTGCAGCCATGGCCGCCCAGACGACACGTGCCCCTGCGGCCTCCCGCACATTCCTGGGGCGCCAG

CAAGCCCCACAGAGGACCGTGGTGCGCGCACGTGCCGCTGCAGCCACCTCCACTGTCACACGCAAGGAGGTGCCTCTGGC

GCTGGAGGAGGGGCCAATGCCACTGAACACATTCAACAACAAGAAGCCCTTCATCGCCAAGGTCAAGTCAGTGGAGCGCA

TCGTGGGCCCCAAGGCCACAGGCGAGACCTGCCACATCATCATTGAGACAGGCGGCGACATCCCATTCTGGGAGGGGCAG

TCATACGGTGTCATCCCACCAGGCACCAAGGTGAACAGCAAGGGCAAGGAGGTGCCCCACGGCGTGCGCCTGTACTCCAT

CGCCTCCTCCCGCTACGGCGACAGCTTTGACGGCAAGACCACATCCCTGTGCGTGCGCCGTGCAGTGTACAACGACCCAG

AGACAGGCGAGGAGGTGCCAGCCAAGAAGGGCCTGTGCTCCAACCACCTGTGCGACGCCAAGCCCGGAGATGAGATCATG

ATGACCGGTCCCACCGGCAAGATCCTGCTCATGCCAGAGGACAAGAACGCGGTGCTGATCTGCGTGGCCACGGGCACAGG

CATTGCGCCCTACCGCGCGTTCTGGCGCCGCTGCTTCTACGAGGACGTGCCCAACTACAAGTTCAAGGGCCTGTTCTGGC

TGTTCATGGGCGTGGCAAACAGCGACGCCAAGCTGTACGACGACGAGATCCGCGAGCTGAGCGCCACCTACCCCGACCAG

TTCCGCGTCGACTACGCGCTGTCGCGCGAGCAGCAGAACGTGCGCGGCGGCAAGATGTACATCCAGGACAAGGTCGAGGA

GTACAGCGACGAGATCTTCTCGCTGCTCGACAACGGCGCGCACATCTTCTTCTGCGGCCTCAAGGGCATGATGCCGGGCA

TCCTGAGCATGCTCGAGCGCGTGGCCAAGGACAAGGGCATGAACTTCGAGGAGTTCATTGAGAAGTTGAAGCACAACAAC

CAGTGGCACGTGGAGGTGTACTAAGCGAGCGCGCTGCAGCTGCTGCAGCACTCCCATGTGGCTGCTATTCCTTTTCAATC

GGAAAGATGCTGCTGTGACCCGCAGCTCGCAAGCACAAGCAGCCAGTGCATAGCAGTTGTGCACGCGCCGCTGTGGACAC

AAAGCATCCATGCATGGGTATCCTTTGGGTCTGAATGTAATCGAACCAGCCAG

>Cluster-342212.133501 NR

AGAGAAGCCGAACAAGTACGGCAAGCACTGGTGCTGGTGCTTCTGGTCCGTCGAGGTCGAGGTTCTGGACCTCCTCGGGG

CCAAGGAGATCGCCGTCCGGGCATGGGACCAGTCGCTCAACACCCAGCCCGAGAAGCTCATCTGGAACCTCATGTGATGA

TCTCTGTGGCAGGGTATGATGAACAACTGCTGGTTCAAGGTGAAGGTGAACGTGTGCAGGCCGCACAAGGGCGAGATCGG

TCTGGTGTTCGAGCACCCGACGCAGCCAGGCAACCAGGCCGGCGGATGGATGGCGCGGCAGAAGCACATGGAGACGGCGG

AGGCCGCCGCACCGGGTCTCAAGCGCAGCACGTCCACGCCGTTCCTCAATACCTCCGGGGACGCCAAGCAGTTCACCATG

TCAGAGGTGCGCAAGCACGCGTCGCAGGAGTCAGCGTGGATCGTGGTGCACGGCCACGTCTACGACTGCACCAAGTTCCT

CAAGGACCACCCGGGTGGCGCCGACAGCATCCTCATCAACGCCGGGACCGACTGCACCGAGGAGTTCGACGCCATCCACT

CCGACAAGGCGCGCGGCCTCCTCGAGATGTACCGCGTCGGCGAGCTCGTCGTCACCGGATCCGACTACGCCTCCCCGCCC

AGCAGCAGCGCCGACCTCACATCCATCGTCGAGCAGTCCCCTGCTGCAGCCGCGCCGCCCCTGCCGCTGCCGGCTATTTC

CACCATCGCGCTTGCCAACCCGAGGGAGAAGGTGAAGTGCCGGCTCGTGGACAAGAAGAGCCTGTCCTACAACGTGCGAC

TCTTCCGGTTCGCGCTTCCCTCGCCGGACCAGAAGCTCGGGCTCCCCGTCGGGAAGCACGTGTACGTGTGCGCGGCGATC

GGCGGCAAGCTCTGCATGCGCGCGTACACGCCGACGAGCTCCGTCGACGAGGTGGGCCACATCGAGCTGCTCATCAAGGT

CTACTACAAGGACGAGGAACCCAAGTTCCCCGCCGGCGGGCTCATGTCGCAGTACCTTGACTCTCTGCCGCTCGGCGTGG

CCATCGACATCAAGGGCCCCATCGGCCACATCGAGTACGCCGGCCGCGGCGCGTTCGTCCTGAACGGCGGCGAGCGGCGG

CGCGCGCGCCGGCTCGCCATGGTCGCCGGCGGGACGGGGATCACGCCCGTGTACCAGGTGATCCAGGCCGTGCTACGTGA

CCAGCCAGAGGACACCACGGAGATGCACCTGGTGTACGCCAACCGGACGGAGGACGACATCCTCCTCCGCGACGAGCTGG

ACCGGTGGGCCAAGGAGTACCCGGACAGGCTCAAGGTGTGGTACGTGATAGACCAGGTGAAGCGGCCGGAGGACGGGTGG

AAGTACAGCGTCGGCTTCGTCACGGAGGCCATCCTAAGGGAGCACGTACCGGAGGGCACCGACGACACGCTGGCGCTCGC

CTGCGGGCCACCACCGATGATCCAGTTCGCCATCAGGCCCAACCTGGAGAAGCTCAAGTACGACTTGGAAAGTTCCTTCA

TCGCCTTCTAAGTAAGAGCCATACCCACCCTCTCGGTTCTTTCATATTTGACGCCTGTTAGTTCAAAACTAAACTATCCC

ACGTCAAATATGAAAGAACAGAGTGGGTATGTTAGTATGTATATACTTGTGTATCCAAAAACCAGTCGAATTGCCTATGC

CATCTCGTTACATGAGATGGTGTTACGGGGTGGGTTTATAATTAAGATGTACAATTGTACTCTTGCTCTAGTCTTAGAGA

CTAGAACACGTACATAGAGGGTCATATTTTGCATGCCCTCAGAATCTCAAGTTCTGAGGAGGCTGCTTGTTTGTCCTGTA

TACTACTAGCAAGTAACCATTGTTGTTACCCTTGCGACCGGGATATCAGAACATGTACGGTAATTA

>Cluster-342212.131909 NIR

AATGCATGTTTAATTTCACGCTGCTCCTTGCCATTCTGTCCGGACGGAGGGAAGCCAGGACACGGCTCCTCGCCGGCGGT

CGCGGATCCGTGCACGGGCGTCTCGCCTCGCCGACGAGCGAGACCGGCCCTTAAGACGGCTGATGGGACGCCTTCTTGCC

TCGACGGGCAGTTTAGGCTTTAACTAAGTCCACGAGCCTTTCTCAGTCGCGGCCTCCCCCACCTGTTAGCCGCGTAACCT

GAGCTTCCGGGCAAGATTACCAAACTGCCCGGCTCTTGGATGTTTCGGAGGAATGGAAGGGCGCCTCAACCAGCACGCAG

TGGCTGAAAACGGCCAGCTCTGCCTCTGGGTTCTCAGGTGGTGTTACACCAAAACAGCTCACGCGAACGTGGGCAGGATG

GTAATTCTGCATCGGTGGATGGCATTTCGCGCCGCATCTGCCTCCTTGAACACCCCAAGGGCCAAGAAACTGCGCTGCGC

GCCCCCTCCCGCCCCACAATAAACAACCACACGCTCGACACCCATGGCCACCACCGCCACCGGCACAGAACAGCAGCAGA

AGCAGCACCACCGCTGCCAACGCTACCATCCGAACCAGCCATGGCTTCTTCCGCATCCTTGCAGCGTTTCCTCCCGCCCT

CACCGCACGCGGCAGCGTCGTCCCGACGGCGCCCCGCCCGGGCCCGCCCCGTGCAGTGCCAGGCCGTCACCGAGACATCG

GCGTCCTCGCCGCCGGTTGTCTCGGAGGAGCGGCTTGAGCAGAGGGTGGAGCAGCGGAAGGGAGGGTACTGGGTGCTCAA

GGAGAAGTTCCGGACGGGGCTGAACCCGCAGGAGAAGGTGAAGCTGGAGAAGGAGCCCATGGGGCTGTTCATGGAGGACG

GCATCAAGGAGCTCGCCAAGCTGCCCATGGAGGAGATCGACGCCAACAAGCTCTCCAAGGAGGACGTCGACGTCAGGCTC

AAGTGGCTCGGGCTCTTCCACCGCCGCAAGCACCAGTACGGTCGCTTCATGATGCGTCTGAAGCTGCCGAACGGCGTTAC

GACGAGCGAGCAGACGCGGTACCTGGCGAGCGTGATCGATGCCTACGGCGAGAACGGGTGCGCCGACGTGACCACCCGGC

AGAACTGGCAAATCCGTGGCGTCACGCTGCCCGACGTTCCGGCCATCCTCGACGGGCTCTACGCTGTCGGGCTCACCAGC

CTGCAGAGCGGCATGGACAACGTGCGCAACCCGGTTGGAAACCCACTTGCCGGCATCGACCCCGACGAGATCGTGGACAC

GCGCCCGTACACCAACCTGCTCTCCTCCTACATCACTAACAACTCACAGGGGAACCCGGAATTCACCAACCTGCCGAGGA

AATGGAACGTCTGTGTGGTCGGCACGCACGACCTGTACGAGCACCCGCACATCAACGACCTCGCCTACATGCCGGCCATC

AAGGACGGGAAGTTTGGTTTCAACCTGCTGGTTGGCGGGTTCATCAGCCCCAAGAGGTGGGGCGAGGCTCTGCCTCTCGA

CGCATGGGTCCCCGGGGACGACATCCTTGCCGTGTGCGGGGCCGTTCTCAAGGCGTTCAGGGACCTCGGCAACAGGGGCA

ACCGGCAGAAGACGCGCATGATGTGGCTCATCGATGAACTCGGGATCGAAGTATTCCGGTCGGAGGTGGAGAAGAGGATG

CCGAACGGTGTGCTGGAACGCGCCGCGCCGGAGGACCTGATCGACAAGACCTGGCAGAGGCGAGACTACCTGGGCGTGCA

CCCGCAGAAGCAGGAAGGCCTGTCCTACGTGGGACTGCACGTGCCCGTGGGCCGGTTGCAGGCCTCCGACATGTTCGAGC

TCGCGCGCCTCGCCGACGAGTACGGCTCCGGCGAGCTCCGGCTCACGGTGGAGCAGAACATCGTGCTCCCCAACGTGACG

AACGAAAAGATCGACGCTCTGCTCGCCGAGCCGCTGCTGCAGAAGTTCTCGCCGCAGCCGTCGCTGCTGCTCAAGGGCCT

GGTGGCGTGCACGGGCAACCAGTTCTGCGGGCAGGCCATCATCGAGACCAAGATGCGCGCGCTCAACGTGGCGCAGGAGG

TGGAGAAGCGCGTGTCCGTGCCCAGGACCGTGCGCATGCACTTCACCGGGTGCCCTAACAGCTGCGCGCAGGTGCAGGTG

GCGGACATCGGGTTCATGGGCTGCCTGACCAAGGACAGCAACGGCAAGATCGTCGAGGGGGCGGACATCTACGTCGGCGG

CCGCGTCGGCAGCGACTCGCACCTCGCCGACGTGTACAAGAAGTCCGTGCCGTGCGATGACCTGGTGCCTATCGTGGCTG

ACCTCCTCGTGGAAAAGTTCGGGGCCGTGCCGAGGGAGAGGGAAGAAGATGAGGAGTAGGAGCACCGAGCATTGGCTGCT

GCTGTTCCTGTGATCTTCTTTCGTTGACTCCGAGTTGAGTTACCGGCTGTTCGTTGTAAAGTAGACGATGCCTTCTTCC

>Cluster-342212.77099 FD

TCTCCAGCTCCCCAGCCACACAGAGAAAGAGCGTCTAAGCTCCAGCTAGCTTGCAATGGCCGCCGCACTGAGCCTCCGCG

CGCCCTTCTCCCTGCGCGCCGTGGCGCCACCGGCGCCCCGCGTGGCCCTCGCGCCCGCGGCCCTCAGCCTCGCCGCCGCC

AAGCAGGTGCGGGGAGCCAGGCTCCGCGCGCAGGCGACGTACAAGGTGAAGCTGGTGACGCCCGAGGGCGAGGTGGAGCT

GGAGGTCCCCGACGACGTCTACATCCTCGACCACGCCGAGGAGGAAGGGATCGACCTGCCCTACTCCTGCCGCGCCGGCT

CCTGCTCCTCCTGCGCCGGGAAGGTGGTCTCCGGCCAGCTGGACCAGTCCGACCAGAGCTTCCTCGACGACGACCAGGTG

TCCTCCGGCTGGGTGCTCACCTGCGCCGCCTACCCCACCTCCGACGTCGTCATCGAGACACACAAGGAGGAGGAGCTCAC

CGCTTAATTAAGCTCTAATTTCTATGTACTGCCTTTTGCAAGAGAGTTTGATGAACCAAGAGAAATTTCATCACAGTATA

AAAAATCATTCATGCATGAATATGTCAGTAGTGTACCCATGAGACGGGCCGTTCACCACTGTTATTGCCGGCATGAATTC

GCGCTCTCAAAAGAGATTATGAATTAATGTACTTGTTCCTTTTTTTTTTGTAACATGAGAATACCGTACGTGTGTCTTTA

CACATACCTATTAGCTGATGATGAACGTGGGCTCAAATGCATTGTGTATTATGTTGATCGAAATGGATGAGGGCATACGT

ACGTTTAGTAAGGGCCTAGTACTTATGACATGAATATATATAGCACTATTCGCTAATCTGTTGCTATTGATGTGCTACAT

GCTTTTTTTATTTGCTATATCGAAAGAAAAACAGCGCTAAACACTAATCCAGCGCAGACAAGAGTTTGTTTTTGCATATG

TTGTCATCGCGTATGTTGATGCCCTCCTAATATAAGTGTTGTATTGTGGCTAGCAATTTGTTAGTCATACTTTTTTTGTA

GCAAGCCACATTAGACTAATCTTAGGTGTGGCAGTCTTTGGCATGAACCAAATAGGCCCTAAAAAGCTACTGGTGATAGA

GTTACACGCACACCCCCAGCCATTATGGAAAAAAGGAAAAGAAACTCAGTTTTGT

>Cluster-342212.176762 GS2

ATGGCGCAGGCGGTGGTGCCGGCGATGCAGTGCCAGGTCGGGGCGCGGGCCGTCCACCAGGCG

AGGCCGGCCGGCGCGGGCGGTAGGGTGTGGGCCGCCAGCAGGACCGGCCGGGGCACGTCCGGGTTCAAGCTGATGGCCGT

CAGCACGTCGCGCACCGGGGTGGTACCGCGCCTCGAGCAGCTGCTCAACATGGACACCAAGCCGTACACCGACAAGATCA

TCGCCGAGTACATCTGGGTTGGAGGATCTGGAATCGACATCAGAAGCAAATCAAGGACAATAGAGAGACCAGTTGAGGAT

CCCTCTGAGCTGCCTAAATGGAACTATGATGGATCAAGCACAGGGCAAGCTCCAGGAGAAGACAGTGAAGTCATTCTATA

TCCCCAGGCTATTTTCAGGGACCCATTCCGTGGTGGCAACAACATATTGGTTATGTGCGATACCTATACACCAGCTGGGG

TACCCATTCCTACAAACAAGCGTGCCAGGGCTGCAGAAATCTTCAGCGACCCAAAGGTTGTCAGCGAAGTGCCATGGTTT

GGCATTGAACAGGAGTACACTTTGCTCCAAACAGGTGTGAACTGGCCTCTTGGCTGGCCTGTTGGAGGTTACCCAGGTCC

CCAGGGTCCATACTACTGTGCCGTAGGAGCAGACAAATCCTTTGGCCGTGACATATCAGATGCTCACTACAAGGCATGCC

TGTTTGCTGGAATTAACATTAGTGGAACAAATGGGGAGGTCATGCCTGGCCAGTGGGAATATCAAGTTGGACCTAGTGTT

GGTATTGAAGCAGGAGACCACATATGGATTTCAAGATACATTCTCGAGAGAATCACGGAGCAAGCTGGTGTGGTGCTTAC

CCTTGACCCAAAACCAATTCCGGGTGACTGGAACGGAGCTGGCTGCCACACAAATTACAGCACAAAGACCATGCGTGAAG

ATGGTGGATATGAAGCAATCAAGAAAGCAATCCTGAACCTTTCTCTTCGCCATGATTTGCATATTAGTGCATATGGAGAA

GGAAATGAAAGGAGGTTGACAGGGAAACATGAAACGGCTAGCATAGACAGCTTTTCATGGGGTGTGGCAAACCGTGGTTG

CTCTATTCGGGTGGGGAGAGACACTGAGGCAAAAGGAAAAGGCTACCTGGAAGACCGTCGGCCAGCATCGAACATGGACC

CATACATTGTGACATCGCTACTGGCTGAAACTACAATCCTTTGGGAGCCCACC

>Cluster-342212.168289 GS1

CTCGTTGCCCTCGCCGTAGGCGGCAATGTGCTCGGCGTGCCTCTTGCCGAGCTTCTCGATGGCCTTCTTGATCACCTCGT

AGCCACCGGGCTCCCTCATCGACTTGGTACTGTAGTTGGTGTGGGCACCGGCGCCGTTCCAGTCACCCATGATCGGCTTC

GGGTCGAGGGACAGCACAACCCCAGCTATCTCAGTGATCCTCTCGAGGATGTAGCGGGCCACCCAAATCTGGTCACCGGC

AGCTATTCCGACGGACGGGCCGACTTGGAACTCCCACTGCCCTGGCATGACTTCCCCGTTGATGCCGCTGATGTTGATTC

CGGCGTTAAGGCAGGCCTTGTAGTGGGCGTCAACGACGTCGCGTCCAAAGGCCTTATCGGCACCGGCACCACAGTAGTAT

GGTCCCTGAGGACCAGGGTAGCCACCAACAGGCCAGCCAAGGGGCCAGTTGACATCCTTCTGAAGGAGAGTGTACTCCTG

CTCAATACCATACCATGGCTCCTCAGCTACCACATCAGGGTGGCTTAAGATCTTGGCAGCATTGTGCCTCTTGTTAGTGG

GGATTGGCTCTCCTTGTGGCGTGTAGCAGTCGCACATCACAAGGATGTTGTCGCCCCTCCTGAACGGGTCCTTGAAAATG

GCTTGTGGGTAGAGGATGACTTCACTGTCCTCGCCGGGAGCCTGCCCGGTGCTGGAGCCGTCGTAGTTCCACTTGGGCAG

CTGGCTCGCATCAGTGATGGGGCCTTTCACAGTCCTTGCTTTGCTCCTTAGGTCTATGCCTGATCCTCCAACCCAGATGT

ACTCTGCGATGATCATGTCGGTGCAGTCGCTGAGGTTGAGGTTAACGAGATCGGTGAGGTTGGCCAT

>Cluster-342212.179418 Fd-GOGAT

CCTCAGGAACAAGAATCATAAGAGCTTCAGCAGGACTTCTACCGCTCCGTAGCAGTAATTCAGCAGTGCTATCAAGATTT

GCTGAATCCGATGCTTTAGGGTCACCAAATGGACGTATTTCATGCTCACGACCCCGCCATACAGGAGACTGTACTGTGGC

TTCCCTTGATCGCATCCAGTTCAAGTTCCCCTGTATTGTATTAATTTCTCCATTGTGTCCAAGCAACCTCATAGGTTGAG

CAAGAGGCCATCTAGGATTTGTATTTGTACTGTATCTCCGATGATATATGGCAAAAGGAGATTTGTACAGTTCATTCTGA

AGGTCCAAATAGAATTGCCCAAGAACCTCAGATCGGAGCATTCCCTTGTAGACAATGGTTCTGCTTGACAGAGAGCAAAA

ATAAAGTTCATCTGACCAACTTTCAGATTTTGCAGCCCGCTCTATCAGCTTTCGGGAGATGTACAATTCCCTCTCTATGT

CATCAGCATTATCTTCTTTTGCAACTTTTACAAAAACTTGTTCAATATTAGGCATGGTCTCTTTTGCATTGCGACCTACC

ACTGATACATTAAAAGGAACAGCTCTCCACCCAAGAACCTCAAGGCCTTCGTCCTTAAAAACCTTTTCAGTTACAGCTTT

GGCTTCTGCCGTGGACTTCTCATCTTTTGGAAGGAAGACCATGCCTACACCAGTATTCCTTCTGTCAAAGGGAGCAAGTC

CTTGTTTGCTGGCCCAGTCATCAAACAAGTCCCATGGAATACCACTCATCAGTCCTGCCCCATCACCAGAGTCATTGTCT

GCTCCACATCCACCACGGTGCTCCATGCACCCAAGAGCCATAAGAGCATCGCGAACAATGTTGAATGAAGGCTCATTCCT

CAAGTTTGCAACAAACCCAACACCGCAGGCTCCACGCTCAGACAAGATATCGCTCAAATCCGCAACCTCCTGCACGGGCT

TCTCCCGGCGTGCGACGCCGCCGAGCACGGAGCGGGCGGGGAGCGCCCAGGTCCGCCGCGCCGCCGCGAGCGACGACGGG

CCCCGCGCGCGCAGCCTCCGCGCCGCGGCGACCCTCCCCGCGAGCAGCAGCGGCGGCGCGGCGCGGAGCAGCGGGGGCCG

CGCGGCCGCCGTCGCGGCGGGCGAGGGCGCCGCGCGCGGGAGCGTGGCCATGGACAGGCTCTGGCGGCGGCAACGACGAC

GGGCAGCGCGTGATCCGGTTCGGCGGCGACGAGGACGCGCGCGTGATCCGGTTCGGCGGCGGCGGCGGCGGACAAGAGCG

AGCACCGGCCCCGTCTGCCCGGCCGCCTAGCTAATCAACAGCAAACACACCACGCGTCCGCGCGCGACGGTGTGATATAG

CGCGCGGAAGCGGACAAGGGAGGGGATCGTGTGGAGAGGAGGGGAGCGAAGAGGATAGGGCGGGGGCGGGGGCGGGGACG
